# The inherited variations of a p53-responsive enhancer in 13q12.12 confer lung cancer risk by attenuating TNFRSF19 expression

**DOI:** 10.1186/s13059-019-1696-1

**Published:** 2019-05-24

**Authors:** Lipei Shao, Xianglin Zuo, Yin Yang, Yu Zhang, Nan Yang, Bin Shen, Jianying Wang, Xuchun Wang, Ruilei Li, Guangfu Jin, Dawei Yu, Yuan Chen, Luan Sun, Zhen Li, Qiaofen Fu, Zhibin Hu, Xiao Han, Xin Song, Hongbin Shen, Yujie Sun

**Affiliations:** 1 Key laboratory of Human Functional Genomics of Jiangsu Province, Nanjing Medical University, Nanjing, 211126 China; 2 Department of Epidemiology and Biostatistics, School of Public Health, Nanjing Medical University, Nanjing, 211126 China; 3 Department of Cancer Biotherapy Center, The Third Affiliated Hospital of Kunming Medical University (Tumor Hospital of Yunnan Province), Kunming, 650000 Yunnan China; 4 Collaborative Innovation Center for Cancer Personalized Medicine, Jiangsu Key Lab of Cancer Biomarkers, Prevention & Treatment, Cancer Center, Nanjing Medical University, Nanjing, 211126 China; 5 Department of Cell Biology, Nanjing Medical University, Nanjing, 211126 China; 6 State Key Laboratory of Reproductive Medicine, Nanjing Medical University, Nanjing, 211126 China

**Keywords:** Lung cancer, Risk SNP, Enhancer, TNFRSF19, 13q12.12 risk locus

## Abstract

**Background:**

Inherited factors contribute to lung cancer risk, but the mechanism is not well understood. Defining the biological consequence of GWAS hits in cancers is a promising strategy to elucidate the inherited mechanisms of cancers. The tag-SNP rs753955 (A>G) in 13q12.12 is highly associated with lung cancer risk in the Chinese population. Here, we systematically investigate the biological significance and the underlying mechanism behind 13q12.12 risk locus in vitro and in vivo.

**Results:**

We characterize a novel p53-responsive enhancer with lung tissue cell specificity in a 49-kb high linkage disequilibrium block of rs753955. This enhancer harbors 3 highly linked common inherited variations (rs17336602, rs4770489, and rs34354770) and six p53 binding sequences either close to or located between the variations. The enhancer effectively protects normal lung cell lines against pulmonary carcinogen NNK-induced DNA damages and malignant transformation by upregulating TNFRSF19 through chromatin looping. These variations significantly weaken the enhancer activity by affecting its p53 response, especially when cells are exposed to NNK. The effect of the mutant enhancer alleles on TNFRSF19 target gene in vivo is supported by expression quantitative trait loci analysis of 117 Chinese NSCLC samples and GTEx data. Differentiated expression of TNFRSF19 and its statistical significant correlation with tumor TNM staging and patient survival indicate a suppressor role of TNFRSF19 in lung cancer.

**Conclusion:**

This study provides evidence of how the inherited variations in 13q12.12 contribute to lung cancer risk, highlighting the protective roles of the p53-responsive enhancer-mediated TNFRSF19 activation in lung cells under carcinogen stress.

**Electronic supplementary material:**

The online version of this article (10.1186/s13059-019-1696-1) contains supplementary material, which is available to authorized users.

## Introduction

Lung cancer is the most common type of cancer and shows complex pathogenesis and high heterogeneity. Variations in the predisposition to the disease and disease progression in different ethnic groups imply important roles for germline genetic factors in lung cancer pathogenesis [1]. Although genomic alterations discovered in NSCLC have provided valuable clues for understanding the molecular pathogenesis and genetic susceptibility associated with this disease [2–6], the inherited mechanism of lung cancer has not been well understood yet.

The most common genetic variation in humans is single nucleotide polymorphism (SNP). Genome-wide association study (GWAS) has led to an explosion in the identification of SNPs associated with a variety of complex diseases, including breast, colon, lung, and pancreatic cancers [7–12]. Most of these risk SNPs are located in non-coding regions of the genome. At present, more than a dozen lung cancer risk non-coding SNPs have been identified in various ethnic populations and regions of the world [13–15]. Nevertheless, how these specific risk variants contribute functionally to lung cancer susceptibility and pathogenesis remains unclear.

One challenge to understand the biological functions of specific non-coding DNA risk variants is that these variants do not alter the amino acid composition of a protein. In addition, GWAS attempts to “tag” the approximate locations of disease variants and can identify disease-associated alleles with strong linkage disequilibrium (LD) to tagged SNPs, rather than identifying disease-causative SNPs. Many identified disease-associated SNPs may therefore function as genetic markers or indicators, which increase the complexity of elucidating their biological significance.

The ENCODE Project Consortium has revealed that arrays of long-range regulatory elements are interspersed throughout the whole genome, and most are enhancers [16, 17]. Notably, thousands of GWAS variants have been localized to enhancer elements identified through epigenomic profiling studies. GWAS SNPs are usually correlated with enhancer elements marked with H3K4me1, H3K27ac, and H3K4me3 [18–21]. By contrast, only 10–15% are in LD with a protein-coding variant [22, 23]. Moreover, mutations in single or multiple enhancers are responsible for numerous human diseases, including preaxial polydactyly and Hirschsprung’s disease; breast, prostate, and colon cancers; and human autoimmune traits [24–26]. For example, the new studies reported by Gao et al. and Hua et al. showed that a risk SNP resided in an enhancer directly impact on PCTA19 and CEACAM21 gene expression and prostate cancer prognosis [27, 28]. These studies all support a hypothesis that non-coding causal GWAS variants can contribute to common diseases by perturbing enhancer regulatory activity and consequently interfering with the target gene expression.

The newly identified 13q12.12 locus is highly associated with lung cancer risk in the Han Chinese population. The well-replicated GWAS risk SNP rs753955 (A>G) within this locus is situated in the gene desert region, about 150 kb away from the nearest upstream gene, TNFRSF19. The underlying biological effects of the 13q12.12 lung cancer risk locus are unknown.

In this study, we used an integrative strategy of bioinformatics, laboratory experiments, and clinical analyses to investigate the causative mechanism underlying lung cancer susceptibility associated with the 13q12.12 locus. Our in vitro and in vivo data provided evidence that three inherited causal variations rs17336602 (G>C), rs4770489 (A>G), and rs34354770 (A>C) in 13q12.12 contributed to the lung cancer risk by attenuating the p53-responsive enhancer-mediated TNFRSF19 activation. Our findings provided new insight into the understanding of the lung cancer inherited mechanisms.

## Results

### Identification of the active enhancer within the 13q12.12 locus in high linkage disequilibrium with the risk rs753955

**Fig. 1:**
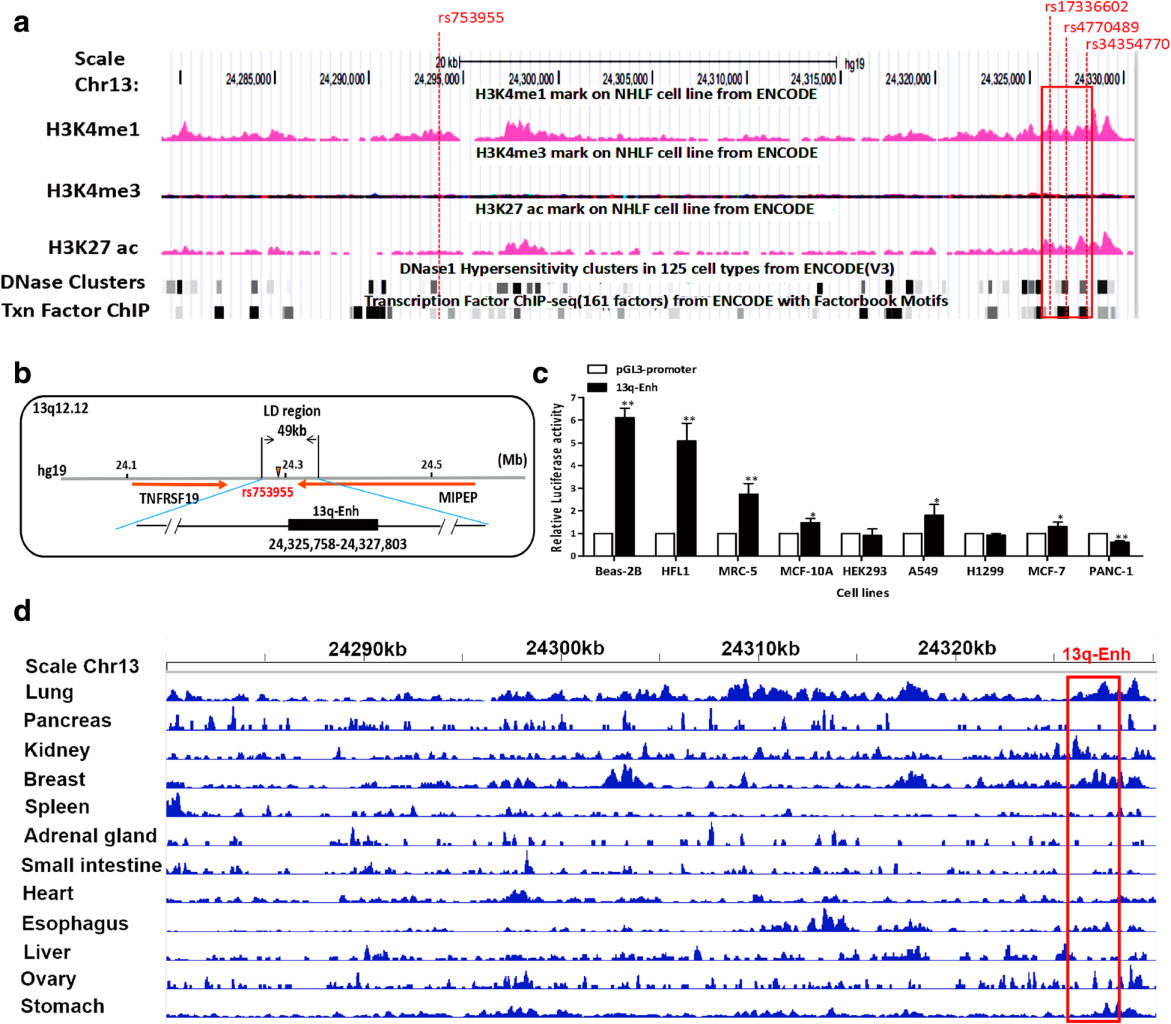
Identification of an active enhancer with lung cell specificity in the high LD region of rs753955. **a** Overview of the epigenetic profiling of H3K4me1, H3K4me3, and H3K27ac chromatin modifications, distributions of DNase I hypersensitivity clusters and transcription factor binding sites in the LD region for the human lung fibroblast cell line NHLF released by UCSC databases. The red rectangle is indicative of the putative active enhancer. The locations of the tag SNP and the three highly linked SNPs within the putative enhancer are indicated by vertical dotted lines in red. **b** A diagram displaying the relative positions of the putative enhancer13q-Enh and two nearest genes, TNFRSF19 and MIPEP, and the Tag-SNP rs753955 marked in a red triangle. **c** Activity and specificity examination of the putative enhancer 13q-Enh. Different cell lines were transiently transfected with the 13q-Enh-pGL3-promoter plasmid and tested for luciferase activity after 24 h. The pGL3-promoter plasmid without the putative enhancer sequence was used as a negative control. The luciferase signal was normalized to the Renilla signal. (*n* = 3 per group; error bars are SD; **p < 0.01, *p < 0.05, unpaired Student’s *t* test). The 13q-Enh displayed significantly high enhancer activity in the normal lung cell lines, Beas-2B, HFL1, and MRC5. **d** The profiling of H3K27ac chromatin modifications of the 13q-Enh region in a lung tissue and 11 non-lung tissues, indicating the high lung tissue specificity of the 13q-Enh activity. The 13q-Enh enhancer region is marked by a red rectangle

We explored the possible mechanisms underlying the lung cancer risk association of GWAS SNP rs753955 (A>G) first by identifying the potential enhancers that functionally contributed to lung cancer risk. HaploView software was used to determine the LD region of rs753955 that spans 49 kb (*r*^2^ > 0.6, *D*′ > 0.9). The putative active enhancers were identified using the data from human lung fibroblast cell line NHLF in the UCSC database based on the criteria including specific histone marks, such as high enrichment of H3K4me1 and H3K27ac and low enrichment of H3K4me3, encompassment of DNase I hypersensitivity, and multiple binding sites of transcription factors (Fig. 1a). Importantly, the putative enhancers should contain common germline genetic variations within or near the transcription factor binding sites, which was confirmed by Variation Viewer in NCBI. One putative enhancer element, namely 13q-Enh, was identified within the 49 kb LD block of rs753955 (Fig. 1a, b).

Subsequently, we tested the regulatory activity and cell type specificity of the enhancer element by cloning the element into pGL3-promoter vectors for luciferase activity tests in different cancer and normal cell lines. Figure 1c showed dramatic enhancer activity displayed by the 13q-Enh element in three normal lung tissue cell lines, Beas-2B human bronchial epithelial cell line, HFL1, and MRC-5 human fetal lung fibroblast cell lines, with 3 to 6 times higher activity than the control, and significantly higher than in other normal tissue cell lines and cancer cell lines. ChIP assays using anti-H3K4me1 and H3K27ac antibodies confirmed the enrichment of H3K4me1 and H3K27ac, the histone marks for active enhancers, on the 13q-Enh (Additional file 1: Figure S1). The tissue specificity of the 13q-Enh enhancer was further evaluated using the available H3K27ac ChIP-seq data for lung and 11 non-lung tissues released by ENCODE database. The 13q-Enh enhancer was rich in H3K27ac in lung tissue. In contrast, the 13q-Enh enhancer was seldom rich in H3K27ac in non-lung tissues except for kidney and breast tissue (Fig. 1d). These in vivo and in vitro data indicated that the 13q-Enh was an active enhancer with high lung tissue specificity.

### Deletion of the 13q-Enh enhancer promoted NNK-induced malignant transformation of Beas-2B bronchial epithelial cells

**Fig. 2:**
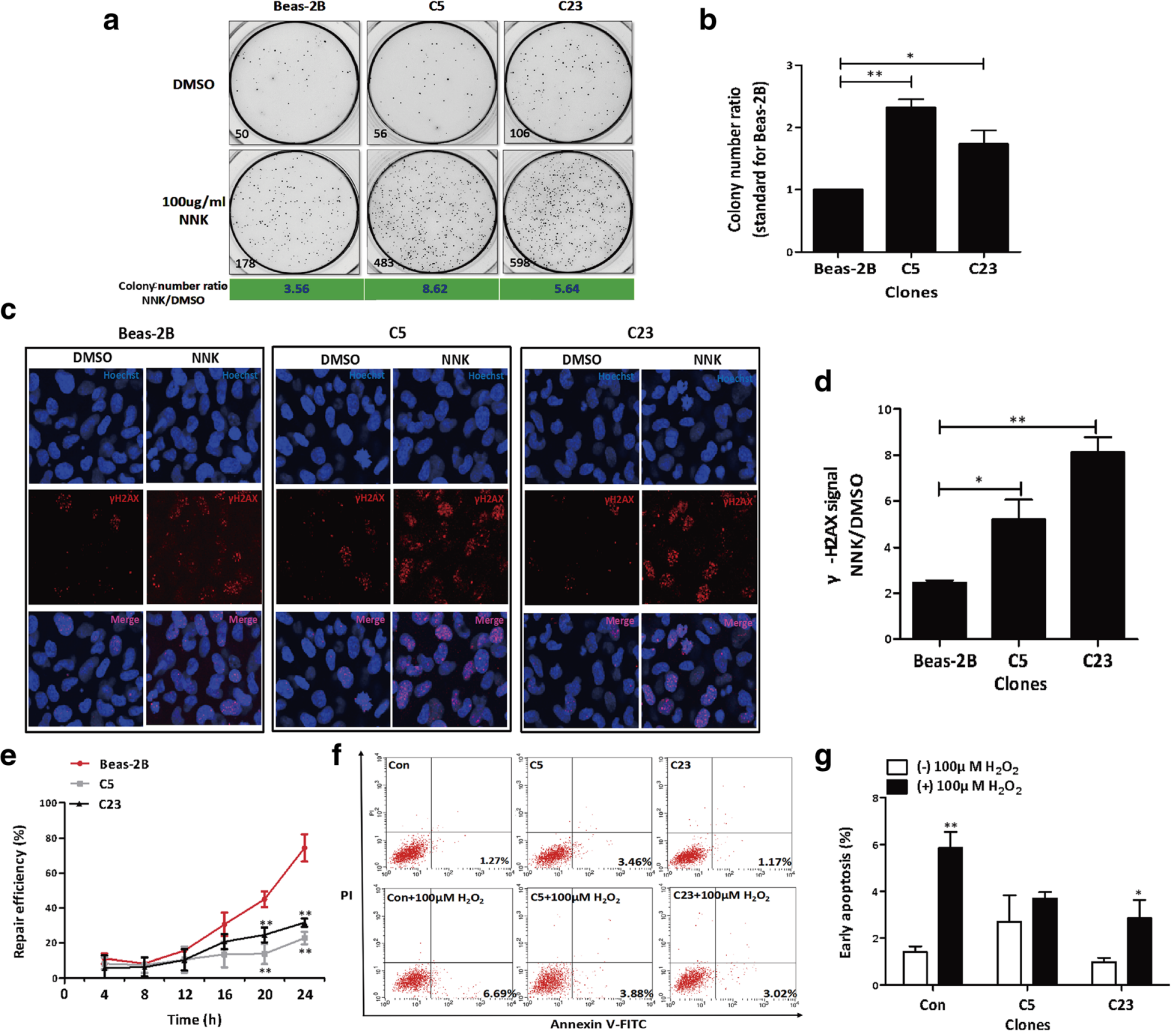
Effects of deleting the 13q-Enh on Beas-2B cells. **a** Soft agar assays for the wild-type and 13q-Enh^−/−^ Beas-2B cells (C5 and C23) treated by NNK for 15 generations. The corresponding colony number is indicated at the lower left corner of each picture. **b** Knockout of the 13q-Enh promoted NNK-induced malignant transformation of Beas-2B cells. The colony number ratios of NNK over DMSO for wild-type and 13q-Enh^−/−^ clones were compared to correct the possible change of “baseline” colony formation caused by knockout of this enhancer. (*n* = 3 per group; error bars are SD; **p < 0.01, *p < 0.05, unpaired Student’s *t* test). **c** γ-H2AX analysis of the cells treated with NNK for 15 generations to evaluate DNA damages in the wild-type cells and 13q-Enh^−/−^ clones. The nuclei were stained with Hoechst 33258 (blue), and breaking DNA ends were detected with γ-H2AX antibody (red). **d** The γ-H2AX signal ratios of NNK over DMSO for the wild-type and 13q-Enh^−/−^ clones were compared. (*n* = 3 per group; error bars are SD; **p < 0.01, *p < 0.05, unpaired Student’s *t* test). **e** DNA repair efficiency of the wild-type Beas-2B cells and the two 13q-Enh^−/−^ clones were compared using host-cell reactivation (HCR) as described in the “Materials and methods” section. DNA repair efficiency was reflected by luciferase activity. The results indicated that deletion of the enhancer significantly impaired the DNA repair efficiency. (*n* = 3 per group; error bars are SD; **p < 0.01, unpaired Student’s *t* test). **f** Cell apoptosis analysis of the H_2_O_2_-treated wild-type Beas-2B and 13q-Enh^−/−^ clones. Cells treated with 100 μM H_2_O_2_ were incubated for 4 h and harvested for Annexin-V/d PI double staining followed by flow cytometry analysis. **g** Statistical analysis of cell apoptosis percentages. (*n* = 3 per group; error bars are SD; **p < 0.01, *p < 0.05, unpaired Student’s *t* test)

We explored the biological functions of the 13q-Enh enhancer by deleting the 13q-Enh enhancer from Beas-2B cells using CRISPR-Cas9 technology, and two 13q-Enh^−/−^ homozygous clones, C5 and C23, were acquired (Additional file 1: Figure S2). We then determined the effects of 13q-Enh deletion on carcinogen-induced malignant transformation. C5, C23, and wild-type control Beas-2B cells were treated with nicotine-derived nitrosamine ketone (NNK), a key tobacco-specific nitrosamine, and a potent pulmonary carcinogen [29], for 15 generations, followed by soft agar colony formation assays (Fig. 2a). The C5-NNK and C23-NNK clones were much more susceptible to NNK-induced cell transformation than the wild-type Beas-2B cells. The transformation rates of the 13q-Enh^−/−^ clones were about twice that of the wild-type cells (Fig. 2b). These data suggested that the 13q-Enh enhancer suppressed carcinogen-induced malignant transformation of bronchial epithelial cells.

### Deletion of the 13q-Enh enhancer reduced DNA repair efficiency and apoptosis responses of Beas-2B human bronchial epithelial cells

Increased DNA damage, together with abnormal apoptosis responses, are well known to contribute to carcinogenesis. We further explored the functions of the 13q-Enh enhancer in cell transformation by using γ-H2AX assays to compare the DNA damage levels in NNK-treated 13q-Enh^−/−^ clones and NNK-treated wild-type Beas-2B cells. Figure 2c and d show significantly increased γ-H2AX signals in the C5-NNK and C23-NNK clones when compared with the NNK-treated wild-type cells, indicating that deletion of the 13q-Enh enhancer increased the sensitivity to NNK-induced DNA breakage. We then used host-cell reactivation (HCR) assays to compare the DNA repair capability of 13q-Enh^−/−^ clones and wild-type Beas-2B cells. The HCR assay is a well-established method to determine the DNA repair capacity of cells. A diagram to explain the principle of the HCR assays was displayed in Additional file 1: Figure S3. Briefly, the pGL3-promoter luciferase plasmids were treated by H_2_O_2_ (*v*/*v*) at room temperature for 1 h to induce DNA breakages. The cells were transfected by the H_2_O_2_-treated plasmids, cultured and harvested at different time points. The plasmids were then purified by ethanol precipitation for luciferase assays. The DNA repair capacity of cells is reflected by the fluorescence curve. The stronger the DNA repair ability of cells, the less the damaged plasmid DNA left in the cells at a certain time point and the stronger the fluorescence value of the reporter plasmids. As shown in Fig. 2e, knockout of the enhancer significantly impaired the DNA damage repair of the cells. The efficiency of DNA damage repair was more than 50% lower in the two 13q-Enh^−/−^ clones compared with the wild-type Beas-2B cells. This clearly implicated that the 13q-Enh enhancer functioned as an important element for maintaining normal DNA repair capability.

We also tested the effects of deletion of 13q-Enh on cell apoptosis. The C5 and C23 clones and the wild-type Beas-2B cells were treated with H_2_O_2_ for 4 h, and subsequent flow cytometry confirmed attenuation of apoptosis in the 13q-Enh^−/−^ clones. The proportion of apoptotic cells decreased from the corrected value of 5.42% in the control to 0.42% and 1.85% in the C5 and C23 clones, respectively (Fig. 2f, g), indicating an involvement of the 13q-Enh enhancer in the regulation of apoptosis.

### The 13q-Enh physically interacted with the TNFRSF19 promoter to upregulate gene expression

**Fig. 3:**
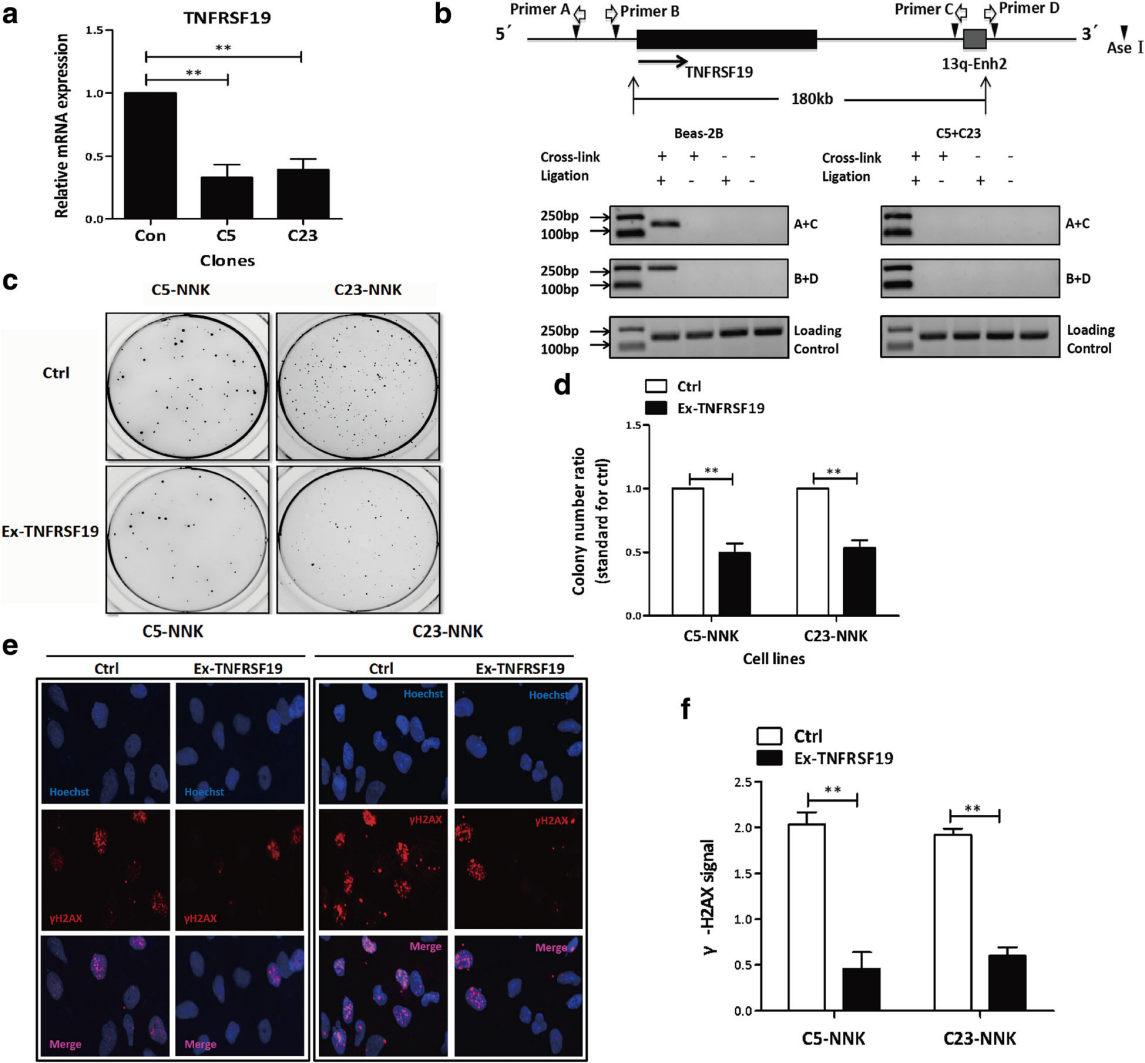
13q-Enh regulated the phenotypes of Beas-2B cells by enhancing the expression of TNFRSF19. **a** The TNFRSF19 expression levels were significantly reduced in 13q-Enh^−/−^ C5 and C23 clones compared with the wild-type Beas-2B cells. (*n* = 3 per group; error bars are SD; **p < 0.01, unpaired Student’s *t* test). **b** A schematic diagram showing the relative positions of the TNFRSF19 gene promoter, 13q-Enh enhancer, *AseI* restriction enzyme cut sites, and PCR primers for 3C assays (upper panel). 3C assays detected the physical interaction between the TNFRSF19 gene promoter and 13q-Enh element in wild-type Beas-2B cells, but not in 13q-Enh^−/−^ clones (a mixture of C5 and C23), either using paired PCR primers, A+C or B+D (low panel). **c** Soft agar assays were performed to determine the effect of restoration of TNFRSF19 expression on NNK-induced malignant transformation in the 13q-Enh^−/−^ clones. The C5-NNK and C23-NNK clones were infected with the lentivirus vectors expressing TNFRSF19 or the empty and were subsequently incubated in soft agar for colony formation. **d** The colony numbers were accounted and statistically analyzed. The results showed that restoration of the TNFRSF19 expression significantly reduced the NNK-induced cell transformation. (*n* = 3 per group; error bars are SD; **p < 0.01, unpaired Student’s *t* test). **e** γ-H2AX was used to evaluate the effects of restoration of TNFRSF19 on the NNK-induced DNA damage in C5-NNK and C23-NNK cells. Cell nuclei were stained with Hoechst 33258 (blue), and breaking DNA ends were detected by γ-H2AX antibody (red). **f** Statistical analysis of the data shown in **e** indicated that restoration of TNFRSF19 expression significantly suppressed NNK-induced DNA damage. (*n* = 3 per group; error bars are SD; **p < 0.01, unpaired Student’s *t* test)

Enhancers generally execute their biological functions by physical interactions with their target genes over a distance through the formation of a chromatin loop. To identify target genes of the 13q-Enh enhancer, we first performed expression quantitative trait loci (eQTL) analysis using the data from the GTEx database and real-time PCR. The eQTL data for lung tissue samples showed that only the TNFRSF19 expression was significantly associated with the risk SNP rs753955 among all genes within the 2-Mbp window of the risk SNP (Additional file 1: Figure S4a & b), suggesting the TNFRSF19 was the potential target gene of the enhancer that would be affected by the genetic variants. Real-time PCR confirmed a reduction of more than 50% in TNFRSF19 expression in the 13q-Enh^−/−^ clones (Fig. 3a). Although the decreased expression of other two genes, SGCG and SACS, were also detected in the 13q-Enh^−/−^ clones, these two genes were not further considered by the present study because of lacking significant association between their expression and the risk SNP rs753955 in lung tissue cells (Additional file 1: Figure S4b & c).

Subsequently, we used chromosome conformation capture (3C) assays to precisely assess the regulation of the 13q-Enh enhancer on TNFRSF19 gene in wild-type Beas-2B cells and 13q-Enh^−/−^ clones. Our 3C assays detected the ligation-dependent PCR products in the wild-type Beas-2B cells, but not in the 13q-Enh^−/−^ clones under the same experimental conditions (Fig. 3b). DNA sequencing further confirmed that the PCR products were derived from the ligation of the 13q-Enh and the TNFRSF19 promoter (Additional file 1: Figure S5), indicating that the 13q-Enh specifically and physically linked with the TNFRSF19 promoter via chromatin looping.

Taken together, these data provided strong evidence that the 13q-Enh enhancer directly targeted TNFRSF19 gene expression by chromatin looping over a span of 180 kb.

### Restoration of TNFRSF19 expression significantly suppressed the NNK-induced cell transformation and DNA damages in 13q-Enh^−/−^ cells

We used rescue experiments to determine whether 13q-Enh executed its biological functions by targeting the TNFRSF19 gene. The 2 13q-Enh^−/−^ clones treated by NNK for 15 generations (C5-NNK and C23-NNK) were transfected with the TNFRSF19-expressing lentivirus or the empty vector control, and the DNA repair efficiency and NNK-induced transformation were examined. As expected, restoring the TNFRSF19 expression significantly suppressed the anchorage-independent growth ability of NNK-treated 13q-Enh^−/−^ clones (Fig. 3c), and the colony formation was reduced by 50% compared with the control (Fig. 3d). Furthermore, γ-H2AX assays confirmed that restoration of TNFRSF19 expression reduced NNK-induced DNA damage in the 13q-Enh^−/−^clones (Fig. 3e, f). These rescue experiments provided evidence that the 13q-Enh enhancer attenuated the risk of carcinogen-induced DNA damage and malignant transformation of bronchial epithelial cells by promoting TNFRSF19 expression.

### The germline genetic variations at rs17336602, rs4770489, and rs34354770 significantly attenuated the enhancer activity by impairing its p53 response

**Fig. 4:**
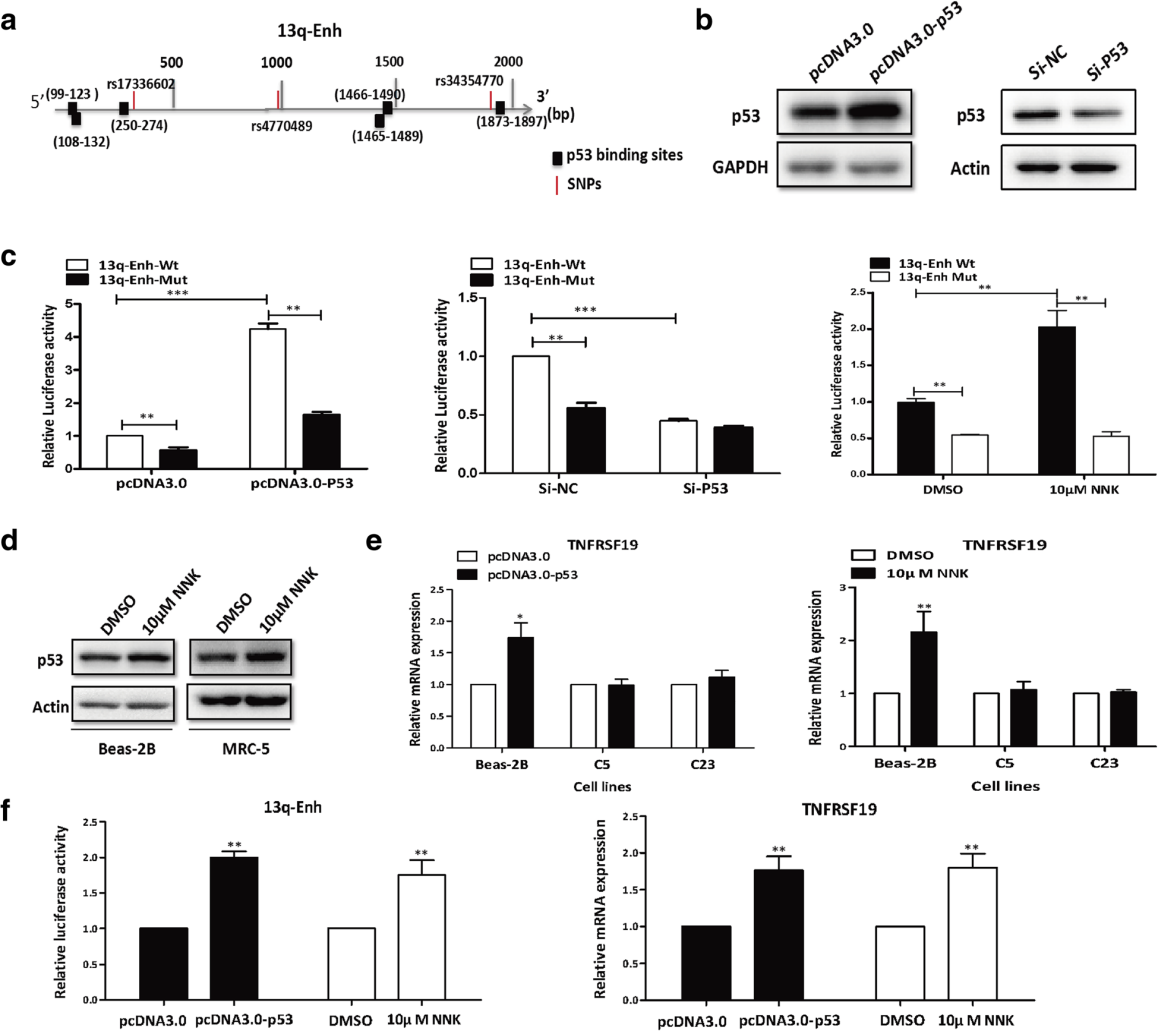
The mutant haplotype reduced 13q-Enh enhancer activity by impairing the enhancer p53 response. **a** Diagram displaying the relative positions of the p53 binding sites and the three common SNPs in the 13q-Enh enhancer. **b** Western blotting confirmed the increased p53 protein level in Beas-2B transfected with p53 expression plasmids (left panel) and the decreased p53 protein level in Beas-2B cells with p53-knockdown (right panel). **c** Luciferase reporter assays showed significantly differential responsiveness of the wild-type allele and the mutant C-G-C allele in Beas-2B cells that either overexpressed p53 (left panel) or knockdown of p53(middle panel) which was stronger than that in the control. So did the responsiveness of wild-type and mutant enhancer in NNK-treated cells (right panel). The mutational effects were strengthened in response to p53 or in the case of NNK exposure. **d** Western blot indicated that NNK treatment significantly induced the expression of p53 in Beas-2B and MRC-5 cells. **e** Real-time PCR revealed that overexpression of p53 significantly increased the TNFRSF19 expression in Beas-2B wild-type cells, but had almost no effect on this gene expression in the 13q-Enh^−/−^ clones (left panel). Similarly, NNK also induced differential expression of the TNFRSF19 in wild-type Beas-2B cells and the 13q-Enh^−/−^ clones (right panel). **f** The 13q-Enh activity (left panel) and the TNFRSF19 expression (right panel) were also significantly enhanced in MRC-5 cells that overexpressed p53 or were treated by NNK

The 13q-Enh enhancer contained three common germline genetic variations, rs17336602 (G>C), rs4770489 (A>G), and rs34354770 (A>C), which were all in high LD with the lung cancer risk SNP rs753955 (A>G) and constituted a haplotype. Notably, bioinformatic analysis using Genomatix software revealed six p53-binding sequences which were either near the three germline genetic variations or between them (Fig. 4a). The mutational effects of these genetic variations on the enhancer activity and p53 response were examined by luciferase reporter gene assays. A mutant 13q-Enh allele that included these three variations was inserted into pGL3-promoter plasmids for the reporter gene assays. As indicated in Fig. 4b and c, left panel, the activity of the enhancer was significantly attenuated by the genetic variations, and alteration of p53 expression confirmed the p53 responsiveness of this enhancer. Importantly, the enhancer p53 response could be impaired by the three genetic variations. The wild-type allele and mutant allele displayed a differential response to p53. The wild-type enhancer greatly responded to p53 expression, while the mutant enhancer had less response (Fig. 4c, left panel). Consistently, in the Beas-2B cells with p53 knockdown, the wild-type enhancer activity was significantly reduced, but the mutant enhancer activity almost stayed at the original low level (Fig. 4c, middle panel). The mutational effect was strengthened when cells were exposed to NNK (Fig. 4c, right panel). Consistent with the responsiveness of the enhancer, the endogenous TNFRSF19 gene also significantly responded to p53 overexpression or NNK treatment in wild-type Beas-2B cells, but not in the clones with deletion of the enhancer (Fig. 4d, e). The enhancer p53 response was well repeated in another lung tissue cell line, MRC-5 (Fig. 4d, f). These data clearly indicated that the 13q-Enh was a p53-responsive enhancer. The three germline genetic variations within the 13q-Enh were able to attenuate the 13q-Enh enhancer activity by impairing the response of the enhancer to p53, especially under the stress of carcinogen exposure.

**Fig. 5:**
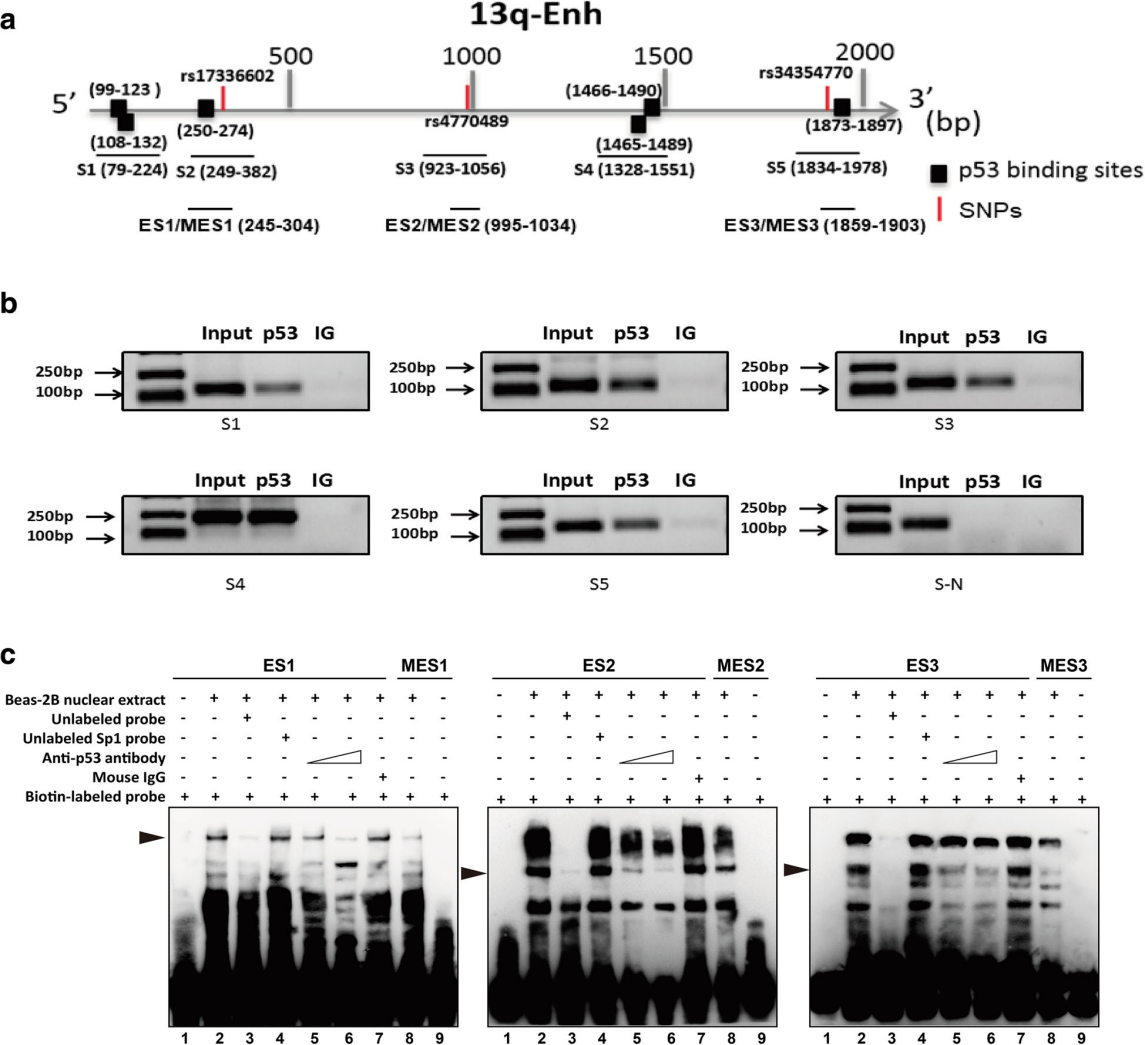
Analysis of p53 bindings on the 13q-Enh enhancer using ChIP assays and EMSA. **a** The schematic diagram showing the relative positions of six p53 binding sites and five sequences for ChIP and three pairs of EMSA probes to the 5′ boundary of the 13q-Enh. **b** ChIP assays showed that all the five sequences, but not the negative control sequence S-N, were specifically precipitated with anti-p53 antibodies, indicating p53 binds to the 13q-Enh in vivo. **c** EMSA assays demonstrated the specific p53 binding to the 13q-Enh and the genetic variations affected the bindings of p53 protein complexes to the enhancer region. Each panel represents the results using the corresponding paired of probes as indicated. Lane 1 and lane 9: negative control using free probes without nuclear extracts; lane 2 and lane 8: using nuclear extract with the biotin-labeled wide-type or mutant probes, respectively; p53 protein complexes formed at wild-type and mutant probes are indicated by black arrows; lane 3: using unlabeled specific wild-type probes pre-incubated with nuclear extracts; lane 4: Sp1 consensus sequence that was used as non-specific control probe; lane 5 and lane 6: using 1 μg and 2 μg anti-p53 antibody to pre-incubate with the nuclear extracts, respectively; lane 7: negative control using 2 μg mouse IgG to pre-incubate with the nuclear extracts

To evaluate the possibility that these variations could affect the p53 binding to impair responsiveness of the 13q-Enh to p53, we first performed ChIP assays to examine p53 bindings on the enhancer in vivo. Bioinformatics analysis predicted five sequences named S1 to S5 that overlap with the potential binding sites of p53 and the three variations we are focusing on in the 13q-Enh region (Fig. 5a). All the five sequences S1–S5 were found to be specifically precipitated with anti-p53 antibodies, while the negative control sequence named S-N (Chr11:108099371-108099488) could not be precipitated with anti-p53 antibodies (Fig. 5b). These ChIP results indicated that p53 bound to multiple sites within the 13q-Enh enhancer in vivo.

The effect of these variations on p53 bindings was subsequently evaluated by electrophoretic mobility shift assay (EMSA) experiments using three wild-type sequences, namely ES1, ES2, and ES3, and the three paired mutated probes, namely MES1, MES2, and MES3, as probes (Fig. 5a). Each pair of the probes contained the potential p53 binding site and the single SNP site (wild type vs. mutant). Several bands were formed when the Beas-2B nuclear extract was incubated with each biotin-labeled wild-type probe. In EMSA using probe ES1, the first band was successfully completed by a 100-fold molar excess of the unlabeled cold probes, but not by cold SP1 consensus sequences, confirming the specific binding of the protein complex to the ES1 sequence (Fig. 5c, left panel, lanes 2–4). Furthermore, the first band was clearly weakened by pre-incubating the nuclear extract with an increasing amount of anti-p53 antibodies, indicating the p53 binding to the ES1 region (Fig. 5c, left panel, lanes 5–6). The genetic variation rs17336602 (G>C) affected the p53 binding, since the band was clearly weakened when Beas-2B nuclear extract was incubated with the biotin-labeled mutant MES1 probe containing this variation under the same experiment conditions (Fig. 5c, left panel, lane 9). The EMSA experiments using the other two pairs of probes, ES2/MES2 and ES3/MES3, also proved that p53 specifically bound to the ES2 and ES3 regions (Fig. 5c, the middle panel, lanes 2–8; right panel, lanes 2–8). The genetic variations rs4770489 (A>G) and rs34354770 (A>C) affected the p53 binding to these two regions as demonstrated by incubating Beas-2B nuclear extract with biotin-labeled MES2 and EMS3 mutant probes containing these two variations, respectively (Fig. 5c, middle panel, lane 9; right panel, lane 9). Our ChIP and EMSA experimental data strongly suggested that the three germline genetic variations impacted the regulatory function of the enhancer by affecting p53 binding to the 13q-Enh enhancer.

### The germline genetic variations at rs17336602, rs4770489, and rs34354770 significantly reduced TNFRSF19 expression in vivo

**Fig. 6:**
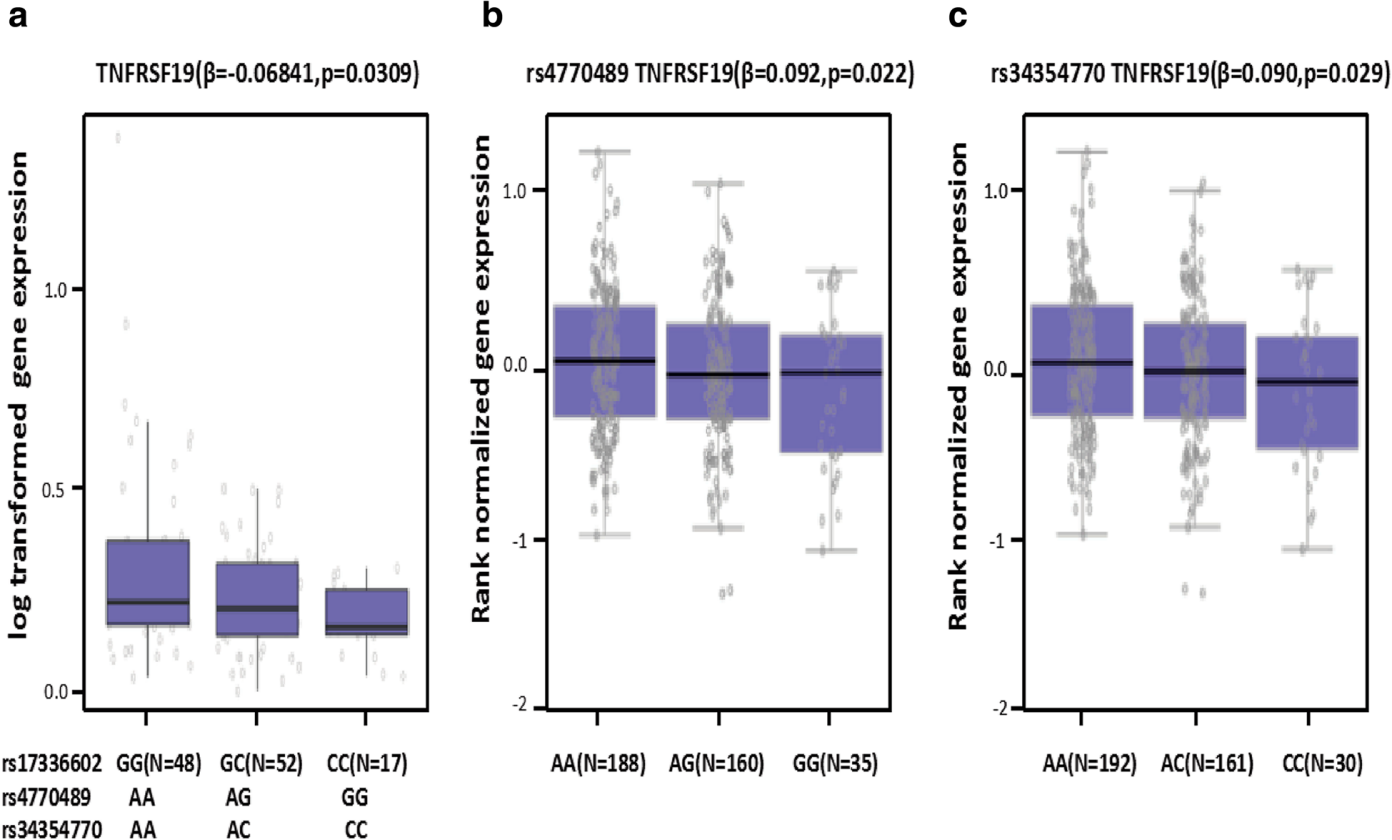
TNFRSF19 expression quantitative trait locus analysis. **a** Genotype and qPCR analyses indicated significant quantitative trait loci (eQTL) associations between genotypes of the mutant haplotype alleles of rs17336602, rs4770489, and rs34354770 with the TNFRSF19 expression in 117 NSCLC samples. TNFRSF19 expression levels were log2 transformed. The box plot displays the first and third quartiles (top and bottom of the boxes) and the median (band inside the boxes). The results are indicated as median and quartiles (*β* = − 0.06841, *p* = 0.031). **b** rs4770489 and **c** rs34354770 eQTL expression analysis with TNFRSF19 using data from the Genotype-Tissue Expression Project (GTEx v7)

Subsequently, we evaluated the impact of the germline genetic variations within the 13q-Enh enhancer on TNFRSF19 target gene expression in vivo through targeted sequencing and real-time PCR analysis of the expression of quantitative trait loci (eQTL) in 117 pairs of lung cancer and para-cancer tissue samples. Figure 6a shows that the germline genetic variations at the 3 highly linked rs17336602, rs4770489, and rs34354770 occurred as a haplotype (C-G-C), and the lung cancer tissues carrying mutant haplotypes expressed significantly lower levels of the TNFRSF19 when compared to the wild-type cancer tissues. The eQTL results from our clinical samples were further supported by the eQTL data for lung tissues from the GTEx database (Fig. 6b, c). These in vivo data supported the notion that the germline genetic variations at rs17336602, rs4770489, and rs34354770 were directly involved in the regulation of the 13q-Enh-modulated TNFRSF19 expression.

### The impacts of deregulated TNFRSF19 expression in vivo and in vitro

**Fig. 7:**
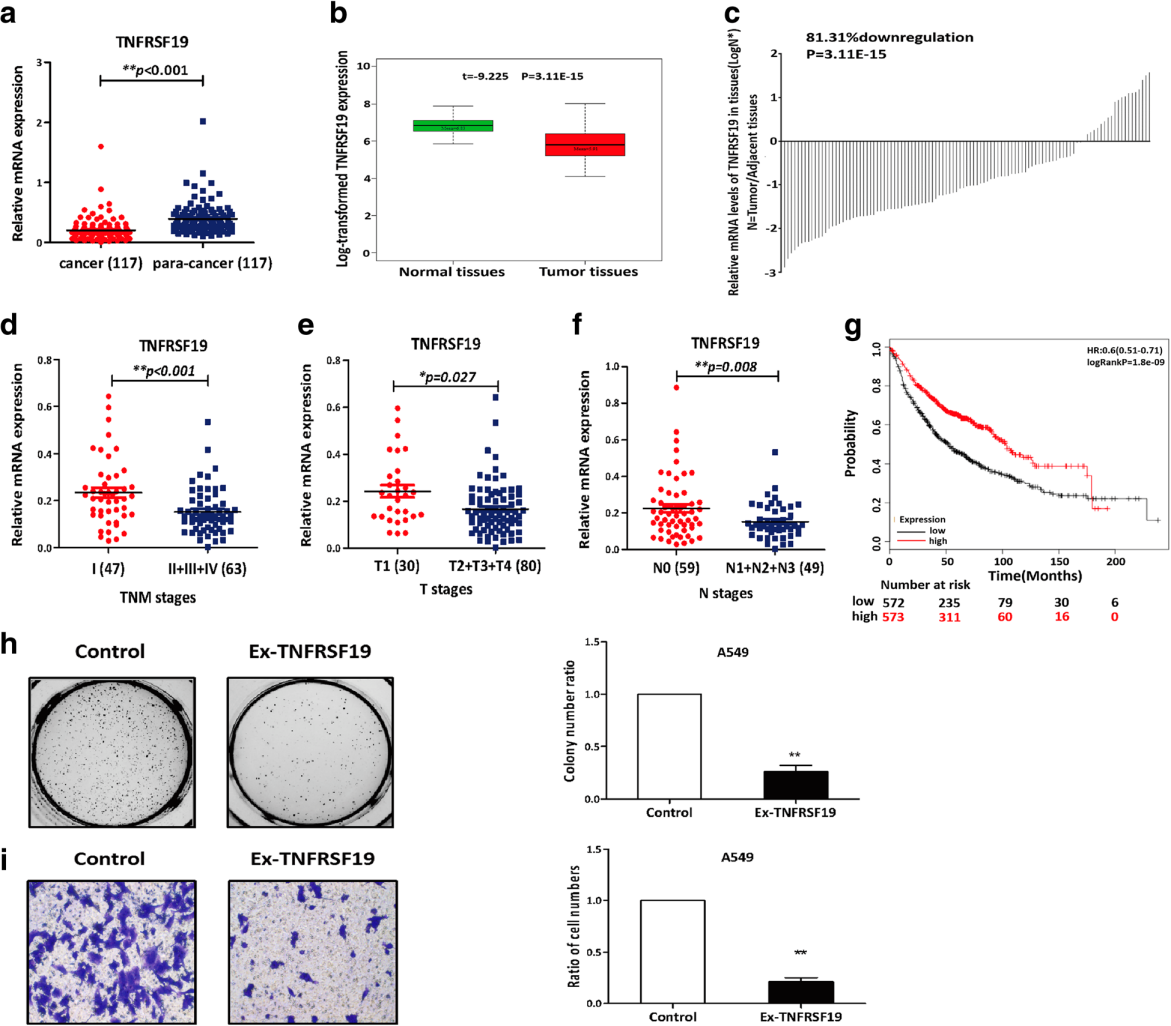
Analysis of the impacts of deregulated TNFRSF19 expression in vivo and in vitro. **a** The expression levels of TNFRSF19 in 117 NSCLC and the paired para-cancer tissue samples were detected by RT-qPCR analyses. The horizontal line indicates the mean expression level. (**p < 0.01, unpaired Student’s *t* test). **b** The expression levels of TNFRSF19 in 107 NSCLC tissues from TCGA on July 8, 2014, and **c** 81.31% showed downregulation of TNFRSF19 expression. The paired sample *t* test was used to examine the differences in gene expression between the tumors and adjacent normal tissues. **d** In total, 110 NSCLC lung cancer samples showed that the TNFRSF19 expression was negatively correlated with tumor staging. The horizontal line indicates the mean expression level. (**p < 0.01, unpaired Student’s *t* test). **e** TNFRSF19 expression was negatively correlated with the tumor size of 110 NSCLC lung cancer samples. The horizontal line indicates the mean expression level. (*p < 0.05, unpaired Student’s *t* test). **f** Analysis of the expression of TNFRSF19 at different N stages of 108 NSCLC lung cancer samples. The horizontal line indicates the mean expression level. (**p < 0.01, unpaired Student’s *t* test). **g** Kaplan-Meier analysis revealed a positive correlation between TNFRSF19 and overall survival (OS) in 1145 NSCLC patients. **h** A549 lung cancer cells that usually express low level of TNFRSF19 were infected with the lentivirus vectors expressing TNFRSF19 or the empty and were subsequently incubated in soft agar for colony formation (left and meddle panels). The colony numbers were statistically analyzed (right panel). The results showed that the introduction of TNFRSF19 into A549 cells significantly suppressed the colony formation. (*n* = 3 per group; error bars are SD; **p < 0.01, unpaired Student’s *t* test). **i** Transwell assays were used to evaluate the effect of the increase in TNFRSF19 on the invasive ability of the A549 cells (left and middle panels). The statistical analysis showed that the introduction of TNFRSF19 into the A549 cells significantly inhibited the cell invasion. (*n* = 3 per group; error bars are SD; **p < 0.01, unpaired Student’s *t* test)

We assessed the potential clinical impacts of deregulation of the TNFRSF19 expression first by real-time PCR analysis to compare the TNFRSF19 expression levels in 117 cancer tissues and their para-cancer tissues. The TNFRSF19 expression was significantly lower in lung cancer tissues than in the para-cancer tissues (Fig. 7a), in agreement with the data from the TCGA [30, 31], Oncomine [32], and GEPIA [33] databases (Fig. 7b, c; Additional file 1: Figure S6a & b). Consistently, the data from the BioGPS database indicated a high and specific expression of the TNFRSF19 gene in the airway and bronchial epithelial cells (Additional file 1: Figure S7) [34]. These data suggested that the TNFRSF19 was required for normal lung tissue cells and the TNFRSF19 deregulation was supportive of lung carcinogenesis.

We also evaluated the relationship between the TNFRSF19 expression levels and the TNM stages in the 117 lung cancer patients. The TNFRSF19 expression levels were inversely correlated with the tumor staging, with significantly high expression in the tumors at stage I and low expression in the tumors at stages II/III/V (Fig. 7d). More specifically, tumors at the T1 stage expressed significantly higher levels of TNFRSF19 than those at the T2/3/4 stages (Fig. 7e). The same was true for lymph node metastasis (Fig. 7f). However, the follow-up time of these 117 patients was not sufficient to determine a relationship between the survival time and the TNFRSF19 expression, so we turned to a Kaplan-Meier plotter database for the information. The survival analysis of 1145 lung cancer patients for more than 200 months showed the survival time was significantly longer for patients with high expression of TNFRSF19 than with low expression [35] (*p* = 1.8e−09, log-rank test; Fig. 7g). The observations that the TNFRSF19 expression was inversely correlated with tumor staging and positively correlated with patient survival time strongly suggest TNFRSF19 functions as a lung cancer suppressor.

We further experimentally tested the tumor suppressor function of TNFRSF19 by transfecting A549 lung cancer cells that express a low level of this protein with TNFRSF19-expressing lentivirus vectors. As expected, the introduction of TNFRSF19 into A549 lung cancer cells significantly suppressed malignant phenotypes of the cells, including colony formation and invasive ability, which is consistent with the clinic observations (Fig. 7h, i) and supported the notion that TNFRSF19 functions as a lung cancer suppressor.

## Discussion

Our present study systematically explored the biological significance and underlying molecular mechanism behind the 13q12.12 locus that is highly associated with lung cancer risk in the Chinese population. We characterized a novel p53-responsive enhancer with normal lung tissue cell specificity in a 49-kb high linkage disequilibrium block of rs753955. It suppressed carcinogen-induced DNA damage and malignant transformation via directly regulating the TNFRSF19 gene over long distance by chromatin looping. The inherited variations at rs17336602 (G>C), rs4770489 (A>G), and rs34354770 (A>C) that were highly linked with the tag-SNP rs753955 (A>G) significantly weakened the enhancer activity by impairing its p53 responsiveness, empowering the potential lung cancer suppressive TNFRSF19 to decrease its eQTL gene expression. In the clinical, the rs17336602-C, rs4770489-G, and rs34354770-C impact lung cancer progression.

One challenge in revealing causative mechanisms of disease risk SNPs in genomic non-coding regions is to connect a risk allele to a target gene. In this study, we provided strong evidence that the TNFRSF19 was a key target gene of the 13q-Enh p53-responsive enhancer and that the inherited variations at rs17336602 (G>C), rs4770489 (A>G), and rs34354770 (A>C) in 13q12.12 significantly weakened the p53-dependent activity of the enhancer. Therefore, it is reasonable to infer that these SNPs can attenuate the 13q-Enh enhancer-mediated TNFRSF19 activation. The inference is further supported by our eQTL analysis with 117 Chinese NSCLC samples. The eQTL data showed a significant association between these SNPs and the TNFRSF19 gene expression, strongly suggesting the involvement of these SNPs in the regulation of TNFRSF19 in vivo. Further test of this inference by introducing the mutant haplotype in the endogenous locus using CRISPR/Cas9 technology will provide more direct evidence for the inference.

The TNFRSF19 protein (also known as Troy) encoded by the TNFRSF19 gene is a member of the TNF-receptor superfamily. The function of TNFRSF19 has not been well understood. Previous reports have suggested complex and pleiotropic roles of this protein in various cellular contexts [36–38]. TNFRSF19 has been reported to have a function of an oncogene in nasopharyngeal carcinoma and colorectal cancers cells [39, 40], but little is known about the biological roles of the TNFRSF19 in lung tissues. The evidence provided by our present study clearly points to TNFRSF19 as a lung cancer suppressor. First, our NNK-induced transformation experiments demonstrated that homologous deletion of the 13q-Enh enhancer resulted in a dramatic decrease in TNFRSF19 expression and increase in double-strand DNA breaks and malignant transformation, while restoration of the TNFRSF19 expression significantly reversed these phenotypes. Second, the introduction of TNFRSF19 into A549 lung cancer cells dramatically suppressed malignant phenotypes of the cells, including soft agar colony formation and invasive ability. Third, TNFRSF19 expression levels were significantly reduced in lung cancer tissues when compared with the para-cancer tissues. Fourth, the TNFRSF19 expression levels were inversely correlated with the tumor staging and survival time, as revealed by our clinical sample analysis of 117 lung cancer patients and Kaplan-Meier analysis of 1145 lung cancer cases. Furthermore, the TNFRSF19 expression patterns in diverse normal human tissues of 262 adult individuals revealed exclusively high expressions of TNFRSF19 in the lung tissue cells and skin cells (Additional file 1: Figure S7), which strongly implies that this protein is indispensable for the normal phenotypes of human lung tissues after birth [34]. In view of the potential clinical significance of TNFRSF19, the lung cancer suppressor function of TNFRSF19 and the underlying mechanisms are worthy of further investigation in vitro and in vivo. The related study is underway in our laboratory.

The tumor suppressor function of TNFRSF19 observed in the present study is not consistent with the previously published papers [39, 40]. However, considering the high tissue-specific expression pattern of TNFRSF19 in adult tissues, it is not difficult to understand such inconsistency. Probably, whether TNFRSF19 functions as an oncogene or a tumor suppressor may depend on when and where it is expressed.

Although the TNFRSF19 was proved to be a critical target gene of the 13q-Enh and there existed no other potential target gene within the 2-Mbp window of the risk SNP rs753955, the present study has not yet ruled out a possibility that 13q-Enh regulates genes other than TNFRSF19 completely. Further analysis using 4C-seq [41] and TNFRSF19 knockout cells to test this possibility is required and helpful.

The p53 tumor suppressor pathway plays a central role in tumor suppression, and the p53 gene is the most frequently mutated gene in human cancer. Alterations in the p53 pathway also attenuate the function of wild-type (WT) p53 in tumors [42]. Our present study suggests a novel route for attenuation of p53 function, namely, by enhancer mutations that disturb p53-dependent enhancer activity. We confirmed that the 13q-Enh harbored six known p53 motifs situated between or near the three causal SNPs and one atypical p53 binding sequence overlapping with the SNP rs4770489. ChIP assays proved that p53 bound to these sequences in vivo. Importantly, the p53 bindings could be significantly affected by the three SNPs as confirmed by our EMSA experiments. Interestingly, in addition to binding to the four sequences with typical p53 motifs, p53 specifically bound to S3 where there does not appear to be a p53 motif (Fig. 5a, b). This may suggest that the lack of a typical p53 binding motif does not rule out the possibility of p53 binding. There might be other unknown motifs that are beneficial to p53 specific bindings. Consistent with the mutant effects of the three causal SNPs on the p53 bindings, the enhancer displayed allele-dependent differential responses to p53, with a significantly lower response of the mutant C-G-C allele compared with the WT allele. This differential response was strengthened when cells were exposed to NNK. The p53-dependent regulation of the enhancer on the target gene was further confirmed by analyzing endogenous gene response. We show that the endogenous TNFRSF19 significantly responds to NNK treatment in wild-type Beas-2B cells, but not in the clones with deletion of the enhancer. These data support the notion that the three inherited variations are involved in the regulatory role of the 13q-Enh by affecting the p53 binding.

In addition to affecting p53 binding, the possibility that the three causal genetic variants affect other TF binding could not be ruled out. Bioinformatics analysis shows that each of these SNPs overlaps with one or more other predicted binding sites of transcriptional factors as usually observed in the cases of genetic variation sites. Whether or not these variants actually affect the other transcription factor binding remains to be explored. Even so, the mutational effects of the three variants on the p53 binding to the 13q-Enh enhancer are significant without a doubt.

Melo et al. have consistently described p53 binding regions located distantly from any known p53 target gene [43]. Many of these p53-bound enhancer regions show enhancer activity and interact intrachromosomally with multiple neighboring genes to convey long-distance p53-dependent transcription regulation. Thus, p53-responsive enhancers may be distributed widely in the genome, lending credence to investigations that explore the extent to which germline genetic variations impair p53-dependent enhancer functions and consequently confer risk of lung cancer and other human cancers. Investigations of this type of genetic variations are especially meaningful for tumors with wild-type p53.

The observation that allele-dependent differential response to p53 is strengthened by NNK indicates that the causal effects of the risk SNPs could be amplified under a given environmental stress. The three tightly linked causal SNPs described in this study are common, as they all occur with the frequency of 0.41 in the Asian population. Therefore, their contributions to lung cancer risk could be significant in individuals exposed to ambient carcinogens or related environmental stresses. The dynamic penetrance of the causal effects of specific germline genetic variations could also partly explain why cancer-risk SNPs can maintain high frequencies in populations.

At any given GWAS locus, multiple SNPs are often found in LD with the GWAS risk SNP. Fine mapping of the location of the causal SNPs and determination of their causal effects, both as individuals or in synergies, are required to identify the causal mechanisms. In the present study, we mapped three causal risk SNPs within the 13q-Enh enhancer that were in high LD with the GWAS lung cancer risk SNP rs753955. Luciferase reporter gene assays showed that the impact of single variation was relatively moderate compared with that of all three variations together, although the mutant alleles including the single variation had a significantly lower activity when compared with the wild-type allele (Additional file 1: Figure S8). The impact on the enhancer activity was distinctively strengthened when all three variations were included, indicating the three highly linked variations acted cooperatively. Furthermore, the mutational effect of the three combined variations on enhancer activity was significantly enhanced in response to p53 or in the case of NNK exposure compared to the control.

## Conclusion

Our present study reveals the biological significance of 13q12.12 lung cancer risk locus. We provide evidence that the three inherited variations at rs17336602 (G>C), rs4770489 (A>G), and rs34354770 (A>C) in 13q12.12 contribute to the lung cancer risk and development by declining the p53-responsive enhancer-mediated TNFRSF19 activation. Our study gives a novel insight into the understanding of the inherited mechanism of lung cancer. It also implies the 13q-Enh and TNFRSF19 as potential biomarkers for lung cancer risk screening and clinical prognosis.

## Materials and methods

### Cell lines

The Beas-2B human bronchial epithelial cell line was kindly supplied by Professor Chaojun Li (Nanjing University, Nanjing, China). The MRC-5 and HFL1 human fetal lung fibroblast cell lines were kindly supplied by Professor Luo Gu (Nanjing Medical University, Nanjing, China) and Wen Ning (Nankai University, China), respectively. The A549 and H1299 human non-small cell lung cancer cell lines were kindly supplied by Professor Lin Xu (Jiangsu Cancer Hospital, Nanjing, China). HeLa human cervical cancer cell line was supplied by professor Wei De (Nanjing Medical University, Nanjing, China). The PANC-1 human pancreatic cancer cell line, HEK293 human embryonic kidney cell line, MCF-7 human breast cancer cell line, and MCF-10A human breast epithelial cell line were purchased from the American Type Culture Collection (Manassas, VA, USA). The cell lines used in this study were authenticated by STR profiling.

### Patient samples

A total of 117 human NSCLC tissues and their adjacent non-cancerous tissues were collected for DNA and RNA isolation from patients with NSCLC from Tumor Hospital of Yunnan province from January 2014 to June 2016. None of the patients had undergone radiotherapy, chemotherapy, or other anticancer treatment before the surgery. The histological features of all specimens were evaluated by pathologists according to the standard criteria, and the clinicopathological characteristics of 117 NSCLC patients are listed in Additional file 2: Table S1. We also obtained mRNA data for NSCLC tissues from The Cancer Genome Atlas (TCGA) on July 8, 2014. The normalized expectation-maximization (RSEM) read counts were available for 107 paired samples (tumors with adjacent normal tissues). The paired sample *t* test was used to examine the differences in gene expression between the tumors and adjacent normal tissues. Expression quantitative trait loci (eQTL) analysis was first performed in the Chinese samples with a linear regression model, and the results were further replicated using data from the Genotype-Tissue Expression Project (GTEx v7).

### Construction of plasmids

The sequence of candidate enhancers designated as 13q-Enh was cloned into the pGL3-promoter vector (Promega Corporation, Madison, WI, USA) between the 5′-MluI-XhoI-3′ restriction sites upstream of the SV40 promoter. The mutant enhancer 13q-Enh was generated at the rs17336602, rs4770489, and rs34354770 sites by site-directed mutagenesis. The wild-type enhancer allele was G-A-A, while the mutant enhancer allele was C-G-C.

The TP53 and TNFRSF19 CDS sequences were cloned into pcDNA3.0 and p-EGFP-C1 vector, respectively, to generate ectopic overexpression plasmids. All primer sequences are listed in Additional file 2: Table S2. All plasmids were confirmed by sequencing (BGI and Invitrogen).

### RNA extraction and quantitative real-time PCR assay

Total RNA was extracted using Trizol reagent (Invitrogen) according to the manufacturer’s protocol. Five hundred nanograms RNA was reverse-transcribed to prepare cDNA according to Roche manufacturer’s instructions. Quantitative real-time PCR was carried out using the SYBR Green for the detection of PCR products: denaturation, 95 °C for 10 min, followed by 40 cycles of denaturation at 95 °C 15 s, annealing at 60 °C for 15 s, and extension at 72 °C for 15 s. The mRNA level of TNFRSF19 and MIPEP was normalized to β-actin. All the primers are listed in Additional file 2: Table S2.

### RNA interference

Beas-2B cells were transfected with siRNA using Lipofectamine 3000 (Invitrogen, USA) to knockdown p53, and the cells were incubated for 48 h before harvesting for luciferase reporter assays. The siRNA sequences are listed in Additional file 2: Table S3.

### Luciferase reporter assay

Two days after transfection, luciferase assays were performed according to the manufacturer’s instructions (Dual-Luciferase System, Promega), and independent triplicate experiments were run for each plasmid. The pGL3-promoter vector was used as a negative control.

### Chromatin immunoprecipitation assay

ChIP assay was conducted according to the manufacturer (Upstate) of ChIP assay kit. Detailed experimental procedures are described in our previous article [44].

### Electrophoretic mobility shift assays

EMSA were performed essentially as previously describe [44]. Nuclear proteins were extracted from Beas-2B cells using NE-PER™ Nuclear and Cytoplasmic Extraction Reagents (78833, Thermo Fisher Scientific), and then measured using TaKaRa BCA Protein Assay Kit (T9300A, TaKaRa) according to the manufacturer’s instructions. Double-stranded oligonucleotides with or without 5′ biotin-labeled were synthesized (Shanghai Generay Biotech Company, Shanghai, China). The sequences of probes are listed in Additional file 2: Table S3. Electrophoretic mobility shift assay (EMSA) was performed with LightShift™ Chemiluminescent EMSA Kit (20148, Thermo Fisher Scientific) and the anti-p53 antibody (ab1101, Abcam). Briefly, nuclear proteins were pre-incubated with unlabeled probe or anti-p53 antibody in a binding mixture for 10–20 min at room temperature, then incubated with labeled wt-probe or mt-probe for 20 min. The mixtures were electrophoresed in 6% non-denatured polyacrylamide gel, transferred to a nylon membrane (INYC00010, Millipore), and detected biotin-labeled DNA by chemiluminescence.

### CRISPR/Cas-9-mediated genome editing

The 13q-Enh enhancer was deleted by co-transfection of sgRNA plasmids and the Cas9 overexpression plasmid into Beas-2B cells. After confirming the deletion efficiency using primers flanking the 13q-Enh region, the cells were sorted into individual 96-well plates with the Aria II cell sorter (BD Biosciences) and subsequently expanded for further analyses.

### NNK-treated cells

The 13q-Enh^+/+^ mixture clone and 13q-Enh^−/−^ clones were exposed to NNK (DMSO as solvent control) at 100 μg/ml (450 μM) continuously for 30 days to induce malignant transformation. Cells were treated with 10 μM NNK for 24 h or 48 h and harvested for luciferase assay and mRNA expression detection.

### Soft agar assays

For soft agar assays, 2 × 10^3^ cells were seeded in 2 ml 0.2%. The agarose stock was diluted with 2× DMEM; medium was overlaid on a 0.25% agarose base in 6-well culture plates. Colonies were stained after 22–28 days with 5% MTT at 37 °C for 4 h and visually counted.

### Immunofluorescence assay

Cells cultured on chamber slides were fixed with 4.0% paraformaldehyde (PFA) at room temperature for 15 min. Slides were blocked with 2% BSA in PBST (PBS + 0.25% Triton X-100), incubated with antibodies (γH2AX, 1:200) overnight at 4 °C, and then incubated with secondary antibody (1:500) at room temperature for 1 h. After three washes with PBST, the slides were incubated with Hoechst (Invitrogen) at room temperature for 20 min. Images were obtained with a FV1000 confocal microscope (Olympus, Center Valley, PA). The fluorescence intensity was determined using ImageJ software.

### Host-cell-reactivation assay

The HCR assay was used to measure the DNA repair capacity [45]. The pGL3-promoter luciferase vector was exposed to H_2_O_2_ (*v*/*v*) at room temperature for 1 h. After stimulation, the damaged plasmids were purified by ethanol precipitation. The luciferase assay was then used to measure the DNA repair capacity of 13q-Enh^+/+^ and 13q-Enh^−/−^ cells after transfection. The stronger the DNA repair ability of cells, the less the damaged plasmid DNA left in the cells at a certain time point and the stronger the fluorescence value of the report plasmids.

### Cell apoptosis analysis

Collected cells were stained with 5 μl Annexin V-FITC and 5 μl PI for 15 min at room temperature, and apoptosis was detected by flow cytometry. All the procedures were conducted according to the manufacturer’s instructions (KeyGenBioTech).

### Chromosome conformation capture assay

The 3C protocol was described previously [44]. Briefly, in a total of 10^6^ wild-type or 13q-Enh^−/−^ Beas-2B cells were harvested and crosslinked in formaldehyde at room temperature. After quenching with glycine, the cells were lysed for nucleus isolation. The isolated nuclei were then digested with *ASEI* at 37 °C. After inactivation of the enzyme, the samples were diluted in 700 μl of ligation reaction system with 100 U T4 ligase and incubated at 16 °C overnight. The ligated chromatin was digested with proteinase K and purified by phenol-chloroform extraction. The interactions between TNFRSF19 and 13q-Enh were detected by specific primers and confirmed by DNA sequencing.

### Construction of lentivirus vector for overexpression of TNFRSF19

The expression vector encoding full-length open reading frame of human TNFRSF19 was constructed by synthesis and PCR amplification. Briefly, the synthetic oligonucleotides were spliced into the complete sequence by PCR and validated by sequencing. Then, it was sub-cloned into the Plvx-EF1A-puro expression vector and validated by sequencing. Plvx-EF1A-puro-TNFRSF19, pSPSV-2 packing vector, and pMD2G envelope vector were transferred to 293T cells. After 72 h transfection, the virus supernatant was filtered and stored at − 80 °C for using.

### Western blot analysis

Protein extracts were boiled in SDS loading buffer and then subjected to 8% SDS-polyacrylamide gel electrophoresis and transferred to PVDF membranes (Bio-Rad). Membranes were blocked in 5% milk-TBST (Tris-buffered saline Tween) for 1 h and then incubated overnight with mouse p53 antibody (Santa Cruz Biotechnology) and mouse actin/GAPDH antibody (Bioworld Technology). The membranes were then washed with TBST and incubated with the appropriate secondary antibody. After washing with TBST, the membranes were developed with an ECL detection system.

### Transwell invasion assays

Matrigel (BD) was diluted with serum-free RPMI 1640 to a final concentration of 3 mg/mL and polymerized in Transwell inserts at 37 °C for at least 4 h. 7 × 10^4^ cells were seeded onto the Matrigel in 10% FBS medium while the bottom chambers contain 500 μl of 20% FBS medium. Cells were allowed to invade for 48 h at 37 °C in 5% CO_2_. Three independent experiments were performed, and at least ten random fields were counted per experiment.

### Statistical analysis

Each experiment was performed at least in triplicate. Results are shown as mean value ± standard deviation (SD). Statistical analysis was performed using unpaired Student’s *t* test. A *p* value less than 0.05 was considered statistically significant.

## Additional files

Additional file 1: **Figure S1.** Analysis of H3K4me1 and H3K27ac modifications on the 13q-Enh using ChIP assays in Beas-2B cells. **Figure S2.** Knockout of the 13q-Enh enhancer in the Beas-2B cell line by CRISPR-Cas9 technology. **Figure S3.** A working diagram of the host-cell-reactivation (HCR) assay. **Figure S4.** The eQTL and real-time PCR analyses of genes within the 2-Mbp window of risk SNP rs753955. **Figure S5.** Sequence analysis of the 3C PCR product. **Figure S6.** TNFRSF19 expression in Oncomine and GEPIA database. **Figure S7.** Specific expression of TNFRSF19 in bronchial/airway epithelial cells. **Figure S8.** Mutational effect of the three joint variations on enhancer activity was significantly stronger than the single variation. (DOCX 3315 kb)

Additional file 2: **Table S1.** Clinicopathological characteristics of 117 NSCLC patients. **Table S2.** The primers used in the present study. **Table S3.** The sequences of siRNA and EMSA probes used in the present study. (DOCX 21 kb)
