# Supplementary material for: The inherited variations of a p53-responsive enhancer in 13q12.12 confer lung cancer risk by attenuating TNFRSF19 expression
Source: Genome Biol. 2019 May 24;20:103. doi: 10.1186/s13059-019-1696-1 (PMC6533720; doi:10.1186/s13059-019-1696-1)
Supplement: Supplementary file 1 — Figure S1. Analysis of H3K4me1 and H3K27ac modifications on the 13q-Enh using ChIP assays in Beas-2B cells. Figure S2. Knockout of the 13q-Enh enhancer in the Beas-2B cell line by CRISPR-Cas9 technology. Figure S3. A working diagram of the host-cell-reactivation (HCR) assay. Figure S4. The eQTL and real-time PCR analyses of genes within the 2-Mbp window of risk SNP rs753955. Figure S5. Sequence analysis of the 3C PCR product. Figure S6. TNFRSF19 expression in Oncomine and GEPIA database. Figure S7. Specific expression of TNFRSF19 in bronchial/airway epithelial cells. Figure S8. Mutational effect of the three joint variations on enhancer activity was significantly stronger than the single variation. (DOCX 3315 kb) [file 13059_2019_1696_MOESM1_ESM.docx]

**
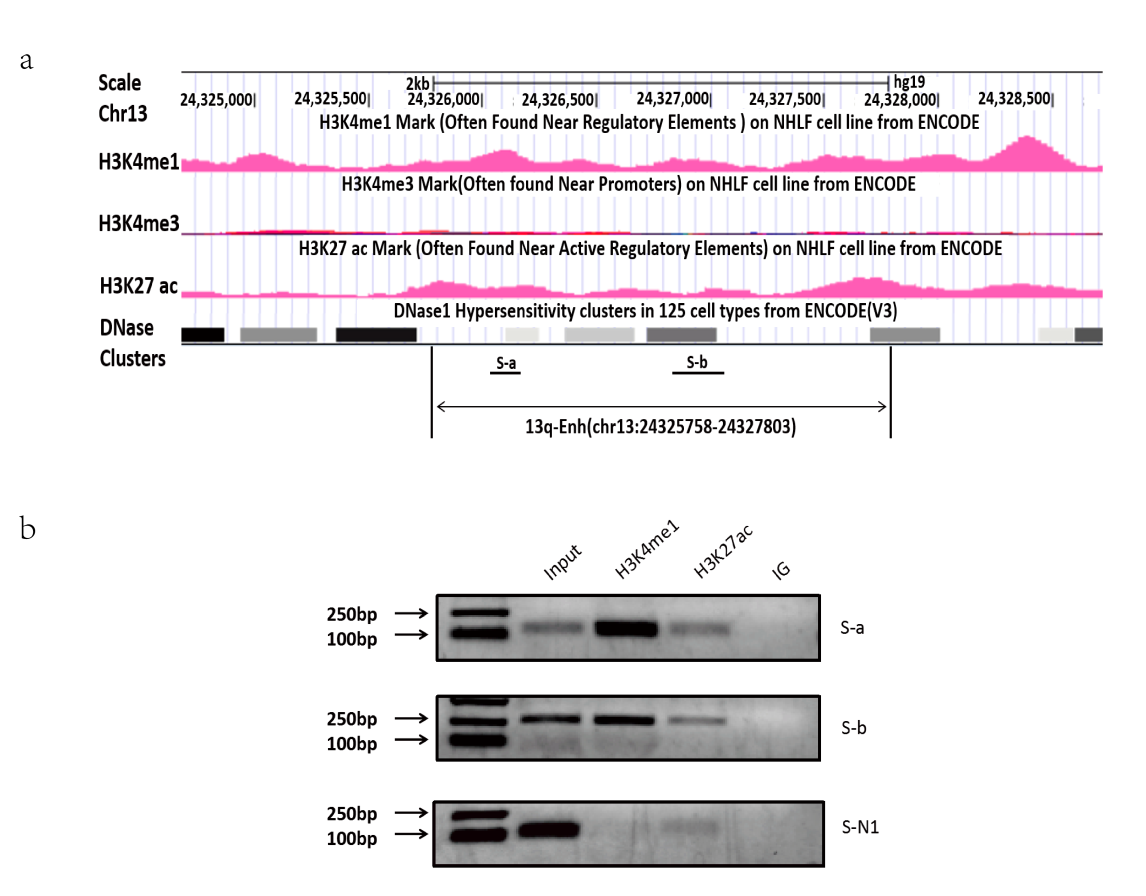
**

**Fig. S1 Analysis of H3K4me1 and H3K27ac modifications on the 13q-Enh using ChIP assays in Beas-2B cells. (a)** A diagram showing the relative positions of S-a and S-b sequences in the 13q-Enh. **(b)** The ChIP assays showed that the both S-a and S-b sequences, but not S-N1 negative control sequence (hg19 chr13:24704575-24704691), were specifically precipitated with anti-H3K4me1 and anti-H3K27ac antibodies (up and middle panels), respectively. confirming H3K4me1 and H3K27ac chromatin modifications in the 13q-Enh. This supported the 13q-Enh as an active enhancer.

**
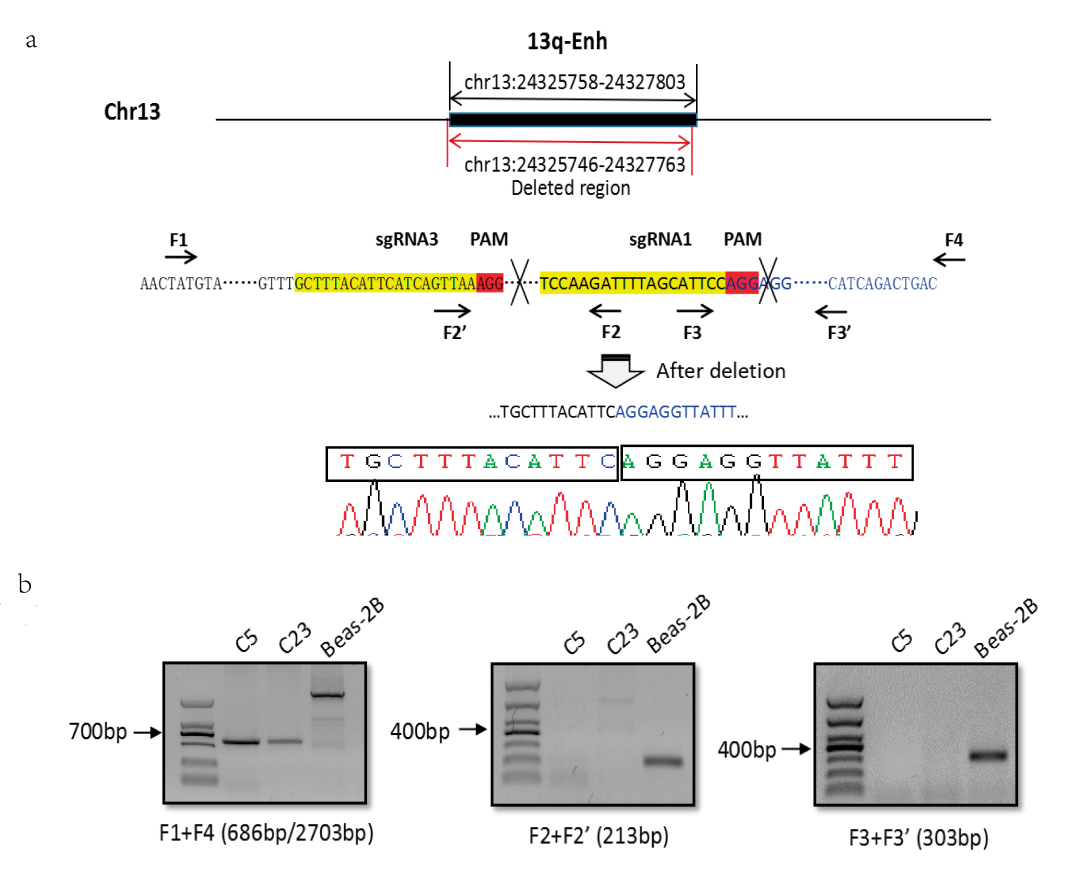
**

**Fig. S2 Knockout of the 13q-Enh enhancer in the Beas-2B cell line by CRISPR-Cas9 technology. (a)**The schematic diagram for knockout of the 13q-Enh by CRISPR-Cas9 technology. PCR primers designed for detecting the bi-allelic deletion are shown as arrows (low panel). **(b)**PCR analysis to determine genotypes of the clones. The long-range PCR in Beas-2B, C5 and C23 cells using primer F1 and F4 showed that C5 and C23 clones presented the sole small band, while wild type Beas-2B cells only presented the large band. These results together with the negative results of PCR using the two pairs of primers F2+F2' and F3+F3' confirmed the bi-allelic deletion in C5 and C23 clones.


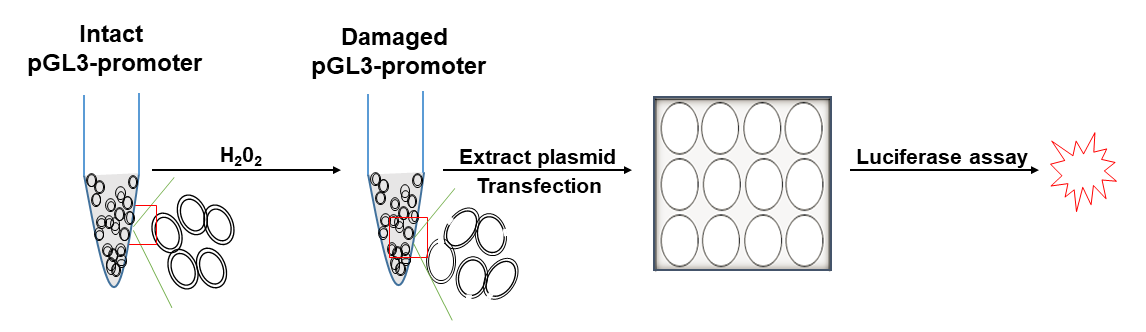


**Fig. S3 A working diagram of the host-cell-reactivation (HCR) assay.** The pGL3-promoter luciferase plasmids were treated by H_2_O_2_ (v/v) to induce DNA breakages.The cells were transfected by the H_2_O_2_-treated vectors, cultured and harvested at different time points. The vectors were then purified for luciferase assays. The DNA repair capacity of cells is reflected by the fluorescence curve. The stronger the DNA repair ability of cells, the less the damaged plasmid DNA left in the cells at the certain time point and the stronger the fluorescence value of the report plasmids.


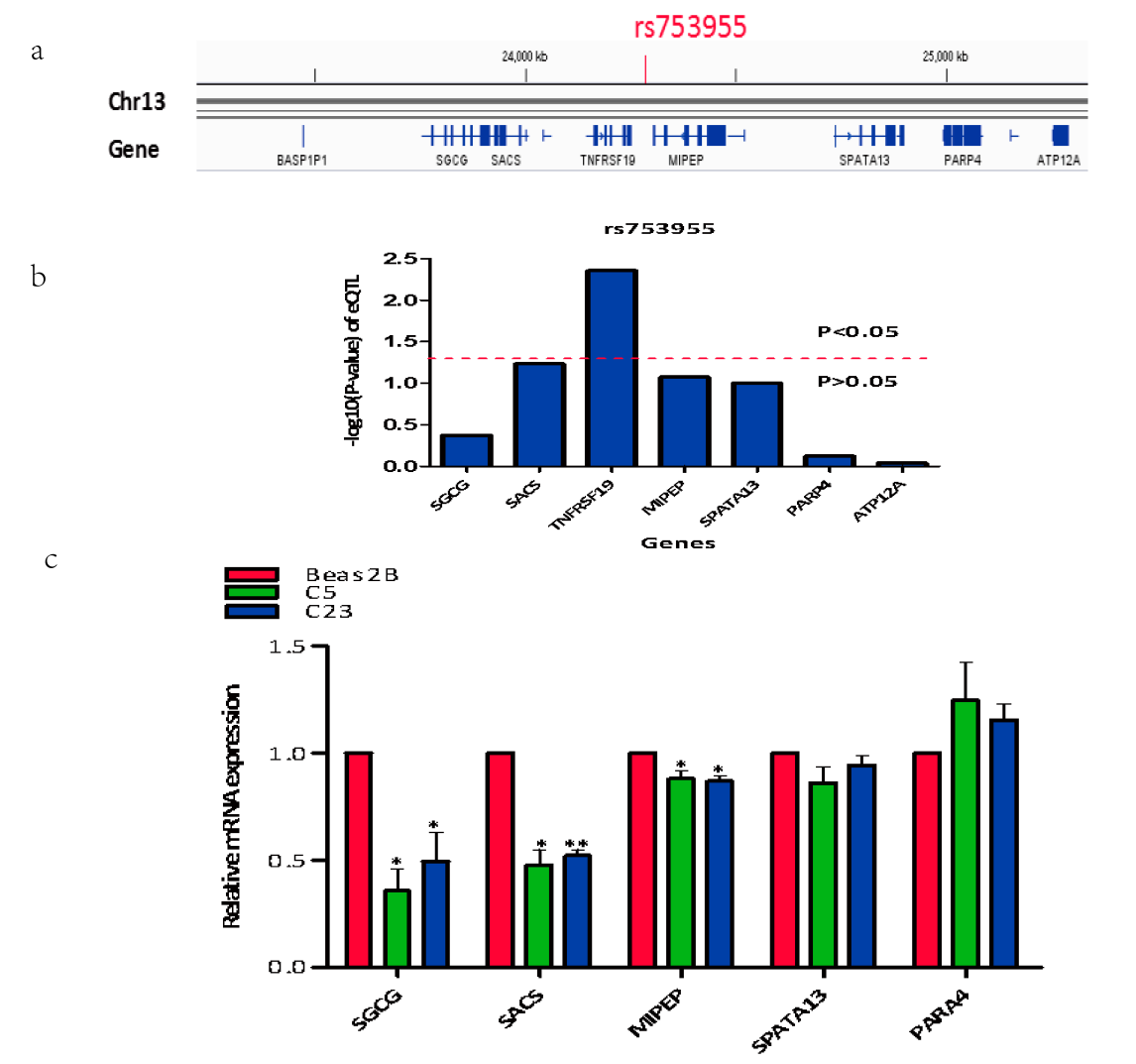


**Fig. S4 The eQTL and real time PCR analyses of genes within the 2-Mbp window of risk SNP rs753955. (a)** A schematic diagram displaying genes within the 2-Mbp window of the risk SNP rs753955. **(b)** The eQTL analysis based on RNA-seq data from lung tissue samples released from GTEx datasets showed that among these genes only the TNFRSF19 expression was significantly associated with the risk SNP rs753955. **(c)** Real time PCR to determine mRNA expression levels of these genes in the wild type Beas-2B cells and the 13q-Enh^-/-^ clones C5 & C23. (n=3 per group; error bars are s.d.; **p < 0.01, unpaired Student's t-test).


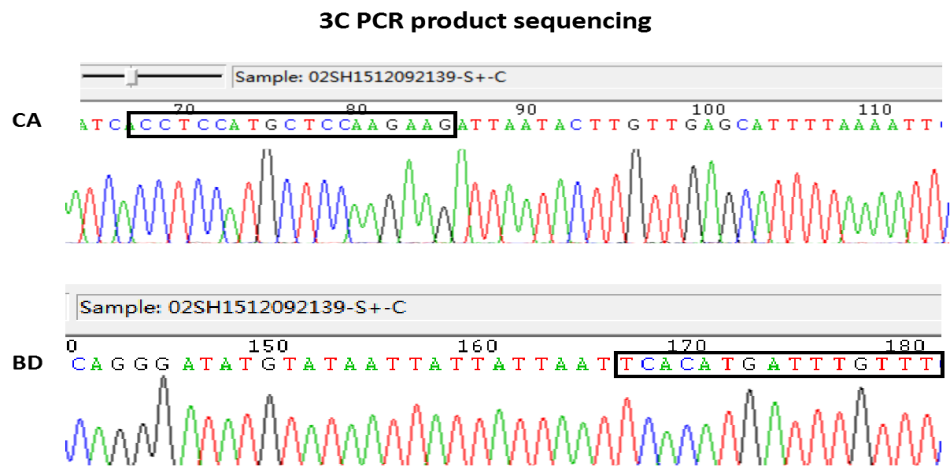


**Fig. S5 Sequence analysis of the 3C PCR product.** 3C PCR product was constructed into a pMD18-T vector (Takara) and sequenced. The black rectangle represents the 13q-Enh region and the rest is the TNFRSF19 promoter region.


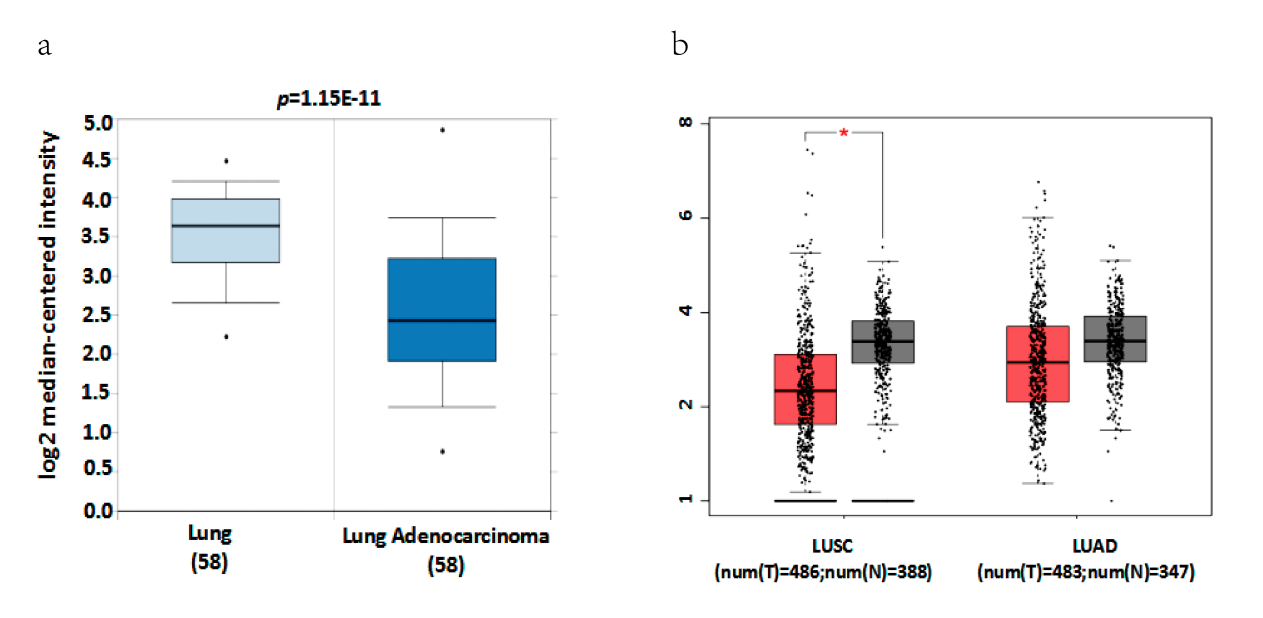


**Fig. S6 TNFRSF19 expression in Oncomine and GEPIA database. (a)** Expression levels of TNFRSF19 in 58 normal lung tissues and 58 lung adenocarcinoma tissues. Expression profile is derived from the Oncomine database. **(b)** Expression levels of TNFRSF19 in 388 normal lung samples, 486 lung squamous cell carcinoma samples, 347 normal lung samples, and 483 lung adenocarcinoma samples. Expression profile is derived from GEPIA database.

**
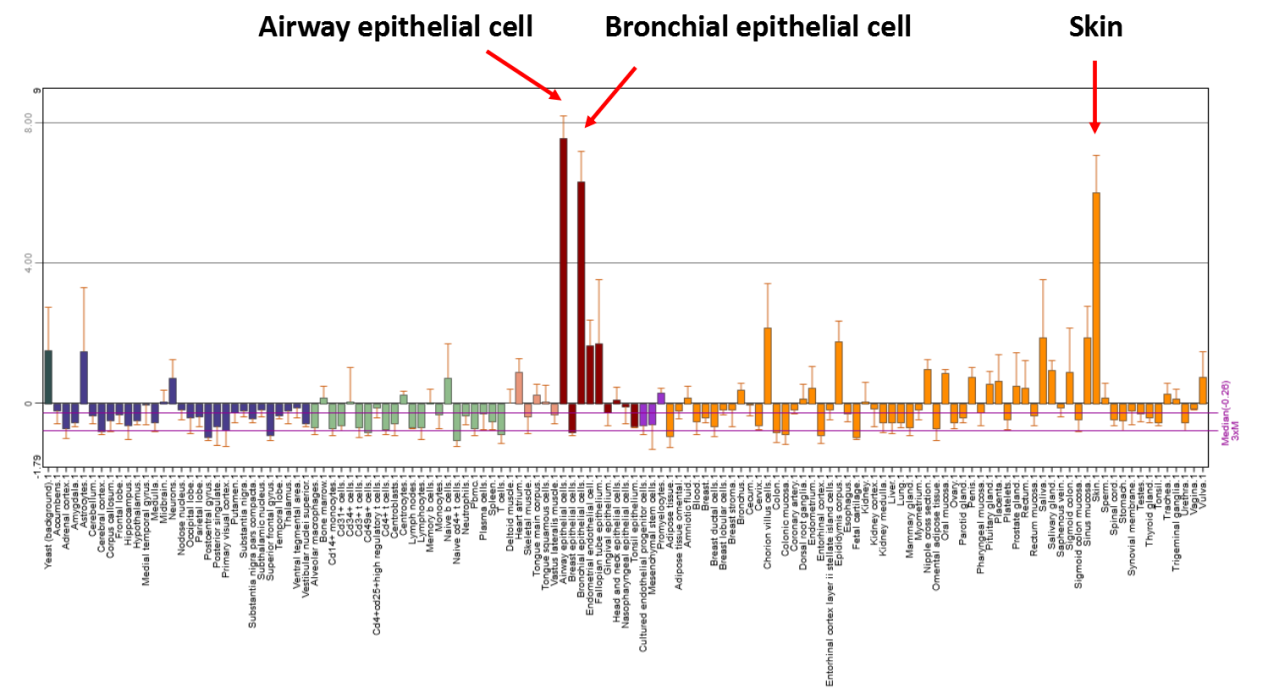
Fig. S7 Specific expression of TNFRSF19 in bronchial/airway epithelial cells.** Dataset in BioGPS displays the expression of TNFRSF19 across diverse normal human tissues from U133plus2.0 Affymetrix microarrays. X-axis represents diverse normal tissues and Y-axis represents the expression level.


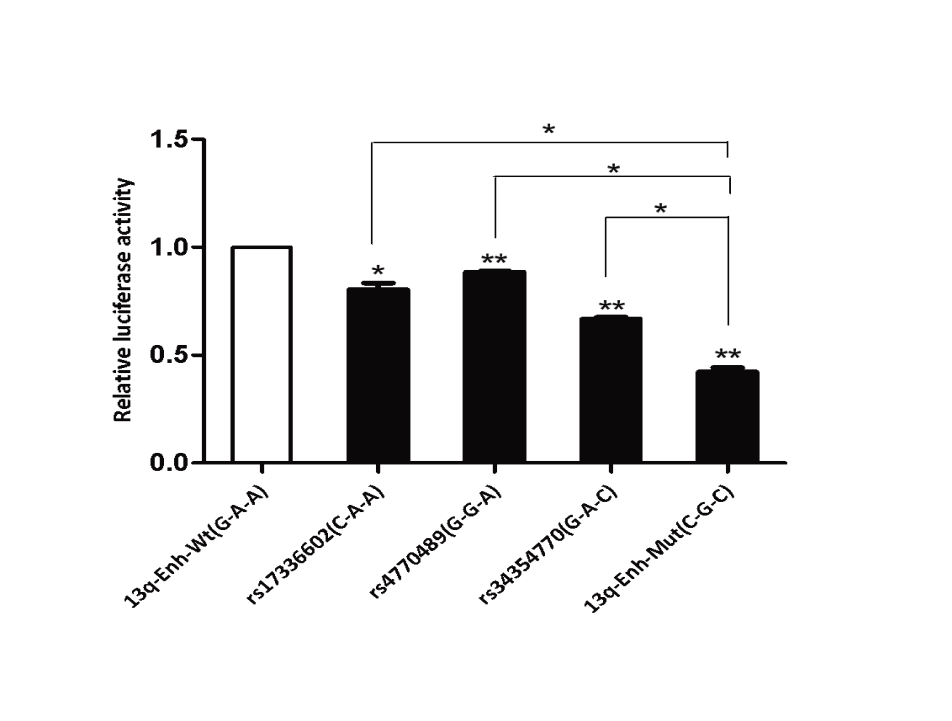


**Fig. S8** **Mutational effect of the three joint variations on enhancer activity was significantly stronger than the single variation.** Luciferase reporter gene assays showed that impairing effect of the single SNP was relatively moderate compared with that of all three variations together, although the mutant enhancer alleles including the single SNP had significant lower activity compared with the wild type allele. The mutational effect on the enhancer activity was distinctively strengthened when all three variations were included.(n=3 per group; error bars are s.d.; *p < 0.05,**p < 0.01, unpaired Student's t-test).
