# Supplementary material for: The inherited variations of a p53-responsive enhancer in 13q12.12 confer lung cancer risk by attenuating TNFRSF19 expression
Source: Genome Biol. 2019 May 24;20:103. doi: 10.1186/s13059-019-1696-1 (PMC6533720; doi:10.1186/s13059-019-1696-1)
Supplement: Supplementary file 2 — Table S1. Clinicopathological characteristics of 117 NSCLC patients. Table S2. The primers used in the present study. Table S3. The sequences of siRNA and EMSA probes used in the present study. (DOCX 21 kb) [file 13059_2019_1696_MOESM2_ESM.docx]

**Table S1. Clinicopathological characteristics of 117 NSCLC patients**

| **Characteristics** | **Number** | **Percent (%)** |
| --- | --- | --- |
| **Age (year)** |  |  |
| < 60 | 62 | 53 |
| ≥ 60 | 55 | 47 |
| **Sex** |  |  |
| Man | 73 | 62 |
| Woman | 44 | 38 |
| **History of smoking** |  |  |
| Ever | 79 | 68 |
| Never | 38 | 32 |
| **Histologic type** |  |  |
| Adenocarcinoma | 82 | 70 |
| Squamous carcinoma | 30 | 26 |
| Other | 5 | 4 |
| **TNM stage** |  |  |
| Ⅰ | 47 | 40 |
| Ⅱ | 27 | 23 |
| Ⅲ | 30 | 26 |
| Ⅳ | 6 | 5 |
| Other | 7 | 6 |

**Other: Information is not found**

**Table S2. Primers used in the present study.**

| **Experiments** | **Primer names** | **Sequences (5’-3’)** |
| --- | --- | --- |
| 3C | Primer A | TGAGTTCGGAAGAAACAGCC |
|  | Primer B | GAAGTGGTCAGTGTTTGAGATG |
|  | Primer C | TACTCCAAAGAATCACCTCCAT |
|  | Primer D | TGACAACTGGCATAACCCTC |
| ChIP assay | S-a-F | ATCAGTGGATGTCAGGCAGGT |
|  | S-a-R | AAGGCAAGAGATGTAAGCAATGT |
|  | S-b-F | AACCTCACCCCAGATGTCTCA |
|  | S-b-R | CCTTGCCTGGCCCAAAGCA |
|  | S-N1-F | CGCTAAACAATGAGTTGAAAGG |
|  | S-N1-R | GAGAAAATGGGAAGAGGTCAGA |
|  | S1-F | CTGTAACCAGAGGCAGAACC |
|  | S1-R | GTGAGCTCCTTTAGACTTTTGTG |
|  | S2-F | ATCAGTGGATGTCAGGCAGGT |
|  | S2-R | AAGGCAAGAGATGTAAGCAATGT |
|  | S3-F | GGTAAAATGAATGGAAATGCC |
|  | S3-R | GGTTTTCTGCTGTTCATCTCATAC |
|  | S4-F | GGGCCAGGCAAGGTGGCTCA |
|  | S4-R | GCAACCTCCACCTCCTGGGTT |
|  | S5-F | GGTATTTGGTGTGTTAGGCTTT |
|  | S5-R | TGTATCTATGCTCTCTAACTCTTGG |
|  | SN-F | CATGCATGCTGTTTTTCCTCTGAT |
|  | SN-R | TGGGTGCATATTTGACCTACCAG |
| Real-time PCR | TNFRSF19-F | GACCTCAGCTCCACGAATATG |
|  | TNFRSF19-R | CACCCCACAACCAAGAGTCG |
|  | MIPEP-F | TGGACCGTGCATGTTCCAC |
|  | MIPEP-R | AGGGTGAGCGATTTTCACAAA |
|  | SGCG-F | GAGCAGTACACTACAGCCACA |
|  | SGCG-R | CGCAGTCCATCTTTTGTTACACA |
|  | SACS-F | GCGCGATGTGAAGGAACGTA |
|  | SACS-R | TCTCCAATCTTGATCCAGTCAGA |
|  | SPATA13-F | CCATTGGGTTGGACCGTGT |
|  | SPATA13-R | GAGGGCTCAGTACCTCCATCT |
|  | PARA4-F | GTGAACAGGATTAGCCTCAACG |
|  | PARA4-R | TCTTAGCCAATAGTCCCAGGTT |
|  | ACTB-F | TCATGAAGTGTGACGTGGACAT |
|  | ACTB-R | CTCAGGAGGAGCAATGATCTTG |
| CRISPR-Cas9 | SgRNA1 | GGAATGCTAAAATCTTGGA |
|  | SgRNA3 | GCTTTACATTCATCAGTTAA |
| Plasmid construction | 13q-Enh-F | ATCTTGGACTTTATCCTTTCCCTTC |
|  | 13q-Enh-R | CCTCTACTTTCTTTTTTCCCTGATG |
|  | pcDNA3.0-p53-F | TTCCACGACGGTGACACG |
|  | pcDNA3.0-p53-R | TCAGTGGGGAACAAGAAGTG |
|  | p-EGFP-TNFRSF19-F | TGGGAAGAACTCTCCAACAATA |
|  | p-EGFP-TNFRSF19-R | TCAGGTTCCCCCAAATCTTA |
| Bi-allelic deletion PCR | F1 | CTGCTTCCTGGATAAATGTA |
|  | F2 | CAGCCAAGAGTTAGAGAGCATAGAT |
|  | F2’ | TGAGTCATCCCTTGGGAACAG |
|  | F3 | GAAAGATTCTCCCCACAGC |
|  | F3’ | TCTGATCTGATGTTTGGTCC |
|  | F4 | TGCGGGCTTCTAGACTTTGG |

**Table S3. The sequences of siRNA and EMSA probes used in the present study**

| **Sequence names** | |  | **Sequences (5’-3’)** |
| --- | --- | --- | --- |
| Si-p53-1 |  | | GTACCACCATCCACTACAA |
| Si-p53-2 |  | | AGAGAATCTCCGCAAGAAA |
| Si-p53-3 |  | | GGAGTATTTGGATGACAGA |
| ES1 | |  | GTTGATCAGTGGATGTCAGGCAGGTCTCTCCAATGTCAATGAGAATAGATAGGTTGCGAC |
| MES1 | |  | GTTGATCAGTGGATGTCAGGCAGGTCTCTCCAATGTCAATGAGAATAGATACGTTGCGAC |
| ES2 | |  | GAAATGTGATTTAACATAAGCCAAAAACCATTGTGGGGGT |
| MES2 | |  | GAAGTGTGATTTAACATAAGCCAAAAACCATTGTGGGGGT |
| ES3 | |  | AGAAAGCCCCTGCTGCTGCAACTTGTCTTTTTTCCTGTGAGGATC |
| MES3 | |  | AGACAGCCCCTGCTGCTGCAACTTGTCTTTTTTCCTGTGAGGATC |
| SP1probe (non-specific  competitive probe) | | | ATTCGATCGGGGCGGGGCGAGC |
